# Widespread Divergence of the CEACAM/PSG Genes in Vertebrates and Humans Suggests Sensitivity to Selection

**DOI:** 10.1371/journal.pone.0061701

**Published:** 2013-04-16

**Authors:** Chia Lin Chang, Jenia Semyonov, Po Jen Cheng, Shang Yu Huang, Jae Il Park, Huai-Jen Tsai, Cheng-Yung Lin, Frank Grützner, Yung Kuei Soong, James J. Cai, Sheau Yu Teddy Hsu

**Affiliations:** 1 Department of Obstetrics and Gynecology, Chang Gung Memorial Hospital Linkou Medical Center, Chang Gung University, Kweishan, Taoyuan, Taiwan; 2 Reproductive Biology and Stem Cell Research Program, Department of Obstetrics and Gynecology, Stanford University School of Medicine, Stanford, California, United States of America; 3 Institute of Molecular and Cellular Biology, National Taiwan University, Taipei, Taiwan; 4 Discipline of Genetics, School of Molecular & Biomedical Science, The University of Adelaide, Adelaide, South Australia, Australia; 5 Department of Veterinary Integrative Biosciences, Texas A&M University, College Station, Texas, United States of America; Ecole Normale Supérieure de Lyon, France

## Abstract

In mammals, carcinoembryonic antigen cell adhesion molecules (CEACAMs) and pregnancy-specific glycoproteins (PSGs) play important roles in the regulation of pathogen transmission, tumorigenesis, insulin signaling turnover, and fetal–maternal interactions. However, how these genes evolved and to what extent they diverged in humans remain to be investigated specifically. Based on syntenic mapping of chordate genomes, we reveal that diverging homologs with a prototypic CEACAM architecture–including an extracellular domain with immunoglobulin variable and constant domain-like regions, and an intracellular domain containing ITAM motif–are present from cartilaginous fish to humans, but are absent in sea lamprey, cephalochordate or urochordate. Interestingly, the CEACAM/PSG gene inventory underwent radical divergence in various vertebrate lineages: from zero in avian species to dozens in therian mammals. In addition, analyses of genetic variations in human populations showed the presence of various types of copy number variations (CNVs) at the CEACAM/PSG locus. These copy number polymorphisms have 3–80% frequency in select populations, and encompass single to more than six PSG genes. Furthermore, we found that CEACAM/PSG genes contain a significantly higher density of nonsynonymous single nucleotide polymorphism (SNP) compared to the chromosome average, and many CEACAM/PSG SNPs exhibit high population differentiation. Taken together, our study suggested that CEACAM/PSG genes have had a more dynamic evolutionary history in vertebrates than previously thought. Given that CEACAM/PSGs play important roles in maternal–fetal interaction and pathogen recognition, these data have laid the groundwork for future analysis of adaptive CEACAM/PSG genotype-phenotypic relationships in normal and complicated pregnancies as well as other etiologies.

## Introduction

Carcinoembryonic antigen cell adhesion molecules (CEACAMs) and pregnancy-specific glycoproteins (PSGs; also known as Schwangerschafts Protein 1) are characterized by an N-terminal immunoglobulin variable domain-like region followed by a varied number of immunoglobulin constant domain-like structures. The prototypic member, CEA (or CEACAM5), was initially discovered as a membrane-anchored tumor antigen [1]. CEACAM subfamily proteins have since been shown to play important roles in the regulation of immune responses, angiogenesis, the differentiation of mammary glands, insulin signaling turnover, tumorigenesis, and metastasis [2], [3], [4], [5], [6], [7], [8], [9], [10], [11], [12]. By contrast, PSGs have been characterized as highly glycosylated major proteins from human syncytiotrophoblasts during pregnancy. High levels of human PSGs have been detected in maternal serum as early as 3 days postfertilization, coinciding with the attachment of the blastocyst to the uterine wall, and were believed to be critical for protecting the semiallotypic fetus from the maternal immune system during pregnancy in, at least, primates [13], [14], [15], [16], [17], .

Whereas the CEACAM/PSG genes were previously considered eutherian-specific, recent studies have shown that homologs with 30–50% protein sequence similarity to human CEACAMs are present in the marsupial opossum and monotreme platypus [20], [21]. Interestingly, the inventory of CEACAM/PSG genes in mammals appeared to vary greatly even among closely related species. For example, whereas mice contain 27 CEACAM/PSG homologs, rats have only 16 counterparts [20], [21]. In addition, earlier studies of nucleotide substitution and the dN/dS ratio in primates and rodents showed that select CEACAM/PSG genes underwent positive or purifying selection [22], [23]. These earlier observations suggested that the duplication/retention of CEACAM/PSG genes could be susceptible to environmental selection, and the process could be similar to the birth, fixation, and loss of adaptive genes such as olfactory receptors and killer-cell immunoglobulin-like receptors (KIRs) in select vertebrates [24], [25], [26]. Because the analysis of nucleotide or amino acid selection across closely related species provides only a rough estimate of genetic variation within a narrow time frame, how unique the evolution of CEACAM/PSG genes in vertebrates remains to be investigated. In addition, the difficulty in analyzing CEACAM/PSG gene evolution was further aggravated by the large variation in gene repertoire among species, which violates various assumptions of statistical methods commonly used in the analysis of gene selection. Accordingly, we hypothesized that an integrative analysis that encompasses a wide time spectrum is needed to better understand the evolution of CEACAM/PSG genes. Here, based on syntenic mapping of chordate genomes and the analysis of genetic variations in humans, we show that CEACAM/PSG genes represent an independent branch of the immunoglobulin superfamily, and were frequently subject to selection by gene–environmental interactions. Importantly, we also found that human CEACAM/PSG locus is enriched with genetic variations, and the PSG gene inventory could range from 12 to 30 copies among individuals, indicating an ongoing selection of these genes in major, geographically separated human populations. Because PSGs and CEACAMs are important for normal pregnancy and immune responses, our study thus provides a framework for further exploration of adaptive genotype–phenotype relationships involving these fast-evolving genes in reproduction, pregnancy complications, and other patho-physiologies in humans.

## Materials and Methods

### Ethics Statement

All animal works were conducted according to the National Institute of Health standards as set forth by the “Guide for Care and Use of Laboratory Animals”. The collection and use of zebrafish were conducted according to Protocol 12806, assigned to Dr. Sheau Yu Teddy Hsu at Dept. of OB/GYN at Stanford University School of Medicine, approved by the Stanford University APLAC. The pufferfish sample was a gift from Dr. Sheau Yu Teddy Hsu. The deceased fish were part of Dr. Hsu’s personal collection of deceased tropical fish, and the work did not involve any live pufferfish. All work related to platypus tissues was done according to the policies of the animal ethics committee at The University of Adelaide. Platypus tissues were obtained from a deceased adult male platypus under the Animal ethics permits AEEC R.CG.07.03 and AEC S-49-200 to Dr. Frank Grutzner.

### Nomenclature, Protein and Genomic Sequence Data

We used the CEACAM classification proposed by Beauchemin et al. (1999) [27] and Kammerer et al. (2007) [22]. If an orthologous relationship to human proteins could not be defined, the proteins were denoted by the generic gene name in the GenBank. In nonmammalian species, all CEACAM family genes were annotated with a Roman letter based on their chromosomal localization.

Genomic and protein sequences of known CEACAM family genes from humans, mice, rats, and dogs as well as the genomes of major model species were obtained from the NCBI databases (ftp://ftp.ncbi.nlm.nih.gov/genomes), the archive!ensembl database (http://may2012.archive.ensembl.org/index.html), or the JGI database, (http://genome.jgi-psf.org). Sequence alignment was carried out using the program MUSCLE (http://phylogenomics.berkeley.edu) and Tcoffee (http://tcoffee.crg.cat/apps/tcoffee/index.html).

### Identification of CEACAM/PSG Family Genes

Genes belonging to the CEACAM/PSG family from different species were first determined by comparing known CEACAM sequences to genomic contigs using the program BLAST and a series of reciprocal pairwise sequence comparisons as well as the program Genscan [28]. Initially, human and mouse CEACAM sequences were compared against the genomes and proteomes of chimpanzee (*Pan troglodytes*), Rhesus monkey (*Macaca mulatta*), bushbaby (*Otolemur garnettii*), mouse lemur (*Microcebus murinus*), dog (*Canis familiaris*), opossum (*Monodelphis domestica*), platypus (*Ornithorhynchus anatinus*), chicken (*Gallus gallus)*, turkey (*Meleagri gallopavo*), zebra finch (*Taeniopygia guttata*), clawed frog (*Xenopus tropicalis)*, West Indian Ocean coelacanth (*Latimeria chalumnae*), medaka fish (*Oryzias latipes*), zebrafish *(Danio rerio)*, pufferfish (*Takifugu rubripes* and *Tetraodon nigroviridis*), stickleback (*Gasteroteus aculeatus*), little skate (*Leucoraja erinacea*), sea lamprey (*Petromyzon marinus*), amphioxus (*Branchiostoma floridae*), and tunicates (*Ciona intestinalis* and *Ciona savignyi*). Unique protein sequences with E<0.0001 were analyzed with additional blast searches against the human nonredundant protein database to detect the best reciprocal hits. In this step, we excluded homologs for other proteins with IgV-like and IgC-like motifs that did not share a common root with mammalian CEACAMs. Due to sequence divergence, the relationship between newly identified homologs and human CEACAMs cannot be determined unambiguously. Based on this observation and earlier studies, we reasoned that CEACAM family genes likely underwent extensive neo-functionalization or sub-functionalization after gene duplication, and that heterotachy incurred by functional divergence led to the difficulty in phylogeny analysis [29]. Therefore, we sought to determine the phylogenetic relationship of novel CEACAM family genes with syntenic mapping.

### Syntenic Mapping: Determination of Orthologous and Co-orthologous Relationships

Chromosomal localization of CEACAM family genes was obtained from the NCBI database. Syntenic maps were downloaded from the Ensembl’s BioMart data mining tool (http://www.ensembl.org/multi/martview) and Genoscope database (http://www.genoscope.cns.fr/externe/English/Projets/Projet_C/data/synteny/TN_HS_SYNT) [30], [31]. The exact locations for human, chimpanzee, Rhesus monkey, bushbaby, mouse lemur, dog, rat, mouse, opossum, platypus, *X. tropicalis*, *O. latipes, D. rerio*, *T. rubripes,* and *T. nigroviridis* (co)-orthologs were also verified by BLAT searches using the UCSC Genome Bioinformatics webserver (http://genome.ucsc.edu/cgi-bin/hgBlat) [32]. We inferred that a pair of duplicates in teleosts would be whole genome duplication (WGD)-derived co-orthologs if they were located on human-*T. nigroviridis* or human-*D. rerio* syntenic chromosomal regions. By contrast, CEACAM found on neighboring loci on the same chromosome were determined to have been derived from tandem duplications. Therefore, the presence of an ancestor for a select group of CEACAMs in the most recent common ancestor (MRCA) of a select lineage was deduced from analyses combining BLAST results and syntenic mapping. However, we cannot exclude the possibility, albeit a low probability, that some teleost homologs found on duplicated syntenic chromosomal regions were not WGD-derived co-orthologs.

### Phylogenetic Analysis

CEACAM orthologs appear to expand quickly and repeatedly in multiple vertebrate lineages, which is similar to what happened with olfactory and vomeronasal receptors [26]. Due to the high divergence of CEACAM protein sequences, it is impossible to obtain a reliable alignment that includes all CEACAM homologs. We resorted to select representative genes from each lineage so that outlier sequences were not included in the multiple sequence alignment. We constructed representative sets of genes from three targeted clades: primate only, mammal only, and fish only. The amino-acid sequences were aligned using MUSLE v3.8 with default settings [33]. The pairwise genetic distances between amino-acid sequences was estimated using the JTT matrix-based method [34]. All ambiguous positions were removed for each sequence pair. The phylogenetic trees were first constructed using the Neighbor-Joining method [35] implemented in MEGA v5.05 [36]. The bootstrap consensus tree was inferred from 1,000 replicates to represent the phylogenetic relationship between sequences of the proteins analyzed [37].

The phylogenetic trees were also inferred by using the Maximum Likelihood method based on the JTT matrix-based model. The tree with the highest log likelihood is presented. The topology of the tree was constructed using proml in Phylip v3.69 [38]. Initial tree(s) for the heuristic search were obtained automatically by applying Neighbor-Join and BioNJ algorithms to a matrix of pairwise distances estimated using a JTT model, and then selecting the topology with superior log likelihood value. The tree is drawn to scale, with branch lengths which were estimated using codeml of PAML [39]. The bootstrap values of trees in which the associated taxa clustered together shown next to the branches were obtained from 100 replicates.

### F_ST_ estimation, Linkage Disequilibrium (LD) Plot, and the Analysis of CNVs and SNPs

The chromosome 19 SNPs were analyzed based on NCBI dbSNP Build 129 (http://www.ncbi.nlm.nih.gov/projects/SNP/index.html). We computed the unbiased estimate of the F_ST_ for gene region SNPs in the HapMap II using the SPSmart server [40]. LD plots for CEACAM/PSG gene regions were generated using the HaploView 4.1 [41]. The LD blocks were determined using an r2 threshold of 0.8. Analysis of CNVs was performed using the International HapMap Project server (http://hapmap.ncbi.nlm.nih.gov/index.html.en) and manual search of individual publications.

### Expression Analysis

All animal works were conducted according to the National Institute of Health standards as set forth by the “Guide for Care and Use of Laboratory Animals”. The collection of platypus and zebrafish tissues was done according to the policies of the animal ethics committee at The University of Adelaide and Stanford University, respectively. Platypus tissues were obtained from a deceased adult male platypus under the Animal ethics permits AEEC R.CG.07.03 and AEC S-49-200 to Dr. Frank Grutzner. Zebrafish tissues were collected according to the Stanford University APLAC Protocol 12806. Pufferfish tissues were a gift from Dr. Sheau Yu Teddy Hsu at Stanford University School of Medicine.

To verify the zebrafish CEACAM family genes in zebrafish, EST clones were obtained from Open Biosystem and analyzed with dideoxy sequencing. Expression analysis was also performed using snap frozen tissues collected from four adult zebrafish *D. rerio* and an adult male platypus *O. anatinus* as well as three deceased adult pufferfish *T. nigroviridis*. Total RNA was extracted using an RNeasy Mini Extraction kit (Qiagen) or the standard Trizol method. One microgram of RNA was reverse-transcribed in a total reaction volume of 20 µl containing 5 mM MgCl2, 10× buffer, 10 mM dithiothreitol, deoxyribonucleotide triphosphates, Poly-T primers, RNAsin 20 U, and 200 U of MuLV enzyme. Samples were incubated for 45 min at 42°C and 3 min in boiling water. PCR-amplification was performed in 20-µl samples containing 2.0 µl of cDNA, 4.6 µl of water, 2 µl of 10x PCR buffer, 1.2 µl of 25 mM MgCl2, 2 µl of 2.0 µM dNTP, 4 µl of forward and reverse primers, and 0.2 µl of Takara Ex *Taq* polymerase (5 U/µl). After 2 min at 94°C, the amplification was carried out with 35 cycles at 94°C for 30 s and 68°C for 240 s.

To verify that the predicted platypus genes were expressed as mRNA and eliminate the possibility of amplification from DNA templates, gene-specific PCR primers were designed based on sequences on two separate exons.

## Results

### Major Expansion of CEACAM/PSG Genes in Primates Occurred after the Separation of Wet Nosed and Dry-nosed Primates

**Figure 1:**
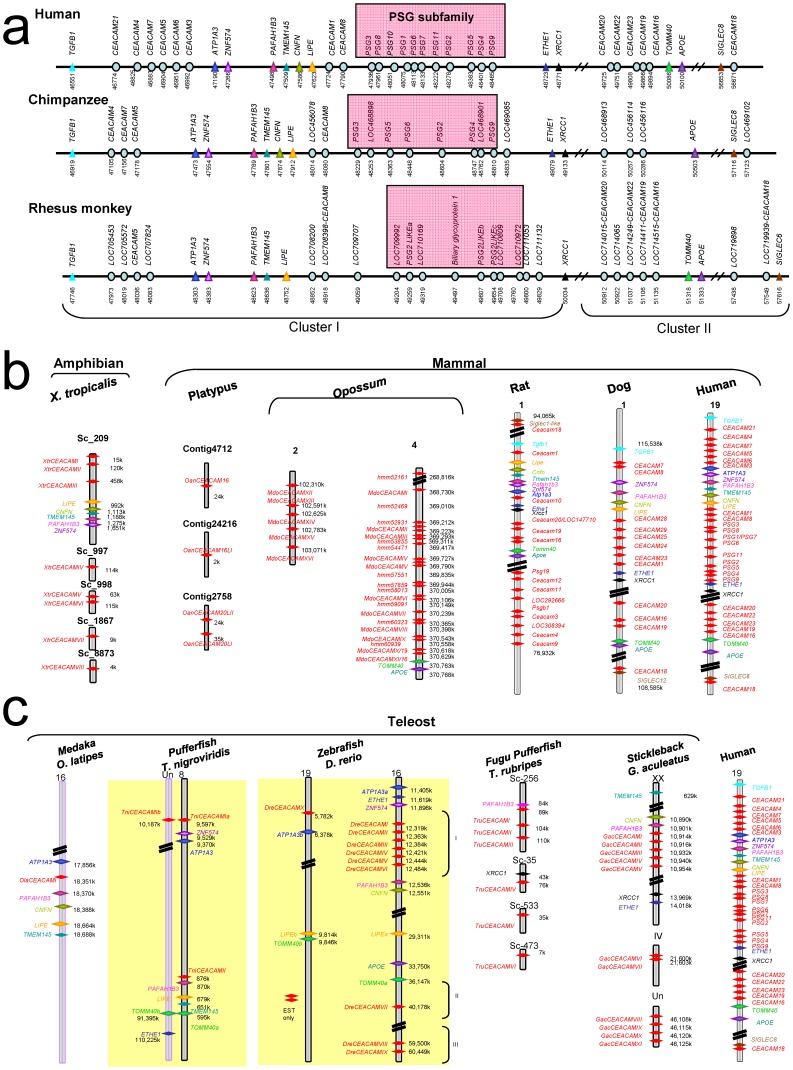
Syntenic mapping of CEACAM/PSG family genes in vertebrates. **a)** Syntenic mapping of the CEACAM/PSG locus in human, chimpanzee (*P. troglodytes*), and Rhesus monkey (*M. mulatta*). CEACAM/PSG genes of these primates can be subdivided into two clusters: cluster I genes are flanked by orthologs of *TGFB1* and *XRCC1* whereas cluster II genes are close to orthologs of *TOMM40, APOE*, and *SIGLEC*8. All CEACAM subfamily genes are indicated by blue oval symbols. CEACAM/PSG locus marker genes, including TGFB1, ATP1A3, ZNF574, PAFAH1B3, TMEM145, CNFN, LIPE, ETHE1, XRCC1, TOMM40, APOE, and SIGLEC8, are indicated by triangle symbols. The PSG subfamily genes are indicated by shaded square boxes. The relative position of genes is shown under gene symbols in Kbp. **b)** Syntenic mapping of CEACAM family genes in dog, rat, the gray short-tailed opossum (*M. domestica*), platypus (*O. anatinus*), and the clawed frog (*X. tropicalis*). The opossum contains more than three dozen paralogs on multiple chromosomes (Table S2 in File S1). For the opossum, only paralogs mapped on chromosomes 2 and 4 are shown. Those found on unknown chromosomes are described in Table S2 in File S1. Among these homologs, eleven (MdoCEACAMI-XI) were found to cluster in a 2-Mbp span on chromosome 4, which also contained the marker genes TOMM40 and APOE. On the other hand, the platypus genome encoded four CEACAM homologs (OanCEACAM16, 16LI, 20LI, and 20LII)(Table S2 in File S1). In *X. tropicalis*, three homologs (XtrCEACAMI-III) were located near marker genes, including LIPE, CNFN, TMEM145, PAFAH1B3, and ZNF574. The chromosomal number and the genomic contig number are indicated at the top of the schematic representation of each genomic fragment. CEACAM family genes are indicated by red diamond-shaped symbols. Marker genes are identified by colored diamond-shaped symbols. The relative position of genes on chromosomes and contigs is shown next to the gene symbols. **c)** Syntenic mapping of CEACAM loci in teleosts. The genomes of the medaka fish (*O. latipes*), stickleback (*G. aculeatus*), zebrafish (*D. rerio*), and two pufferfishes (*T. rubripes* and *T. nigroviridis*) encode 1–12 CEACAM family genes. Syntenic mapping indicated that zebrafish and *T. nigroviridis* CEACAM genes are located on whole genome duplication (WGD)-derived chromosome fragments, and that zebrafish CEACAMs on chromosome 16 are located on three separate loci (I, II, and III). The WGD-derived syntenic chromosomal regions in teleosts are indicated by a yellow background. The chromosomal number and the genomic contig number are indicated at the top of the schematic representation of each genomic fragment. CEACAM family genes are indicated by red diamond-shaped symbols. Marker genes are identified by colored diamond-shaped symbols. The relative position of genes on chromosomes and contigs is shown next to the gene symbols.

**Figure 2:**
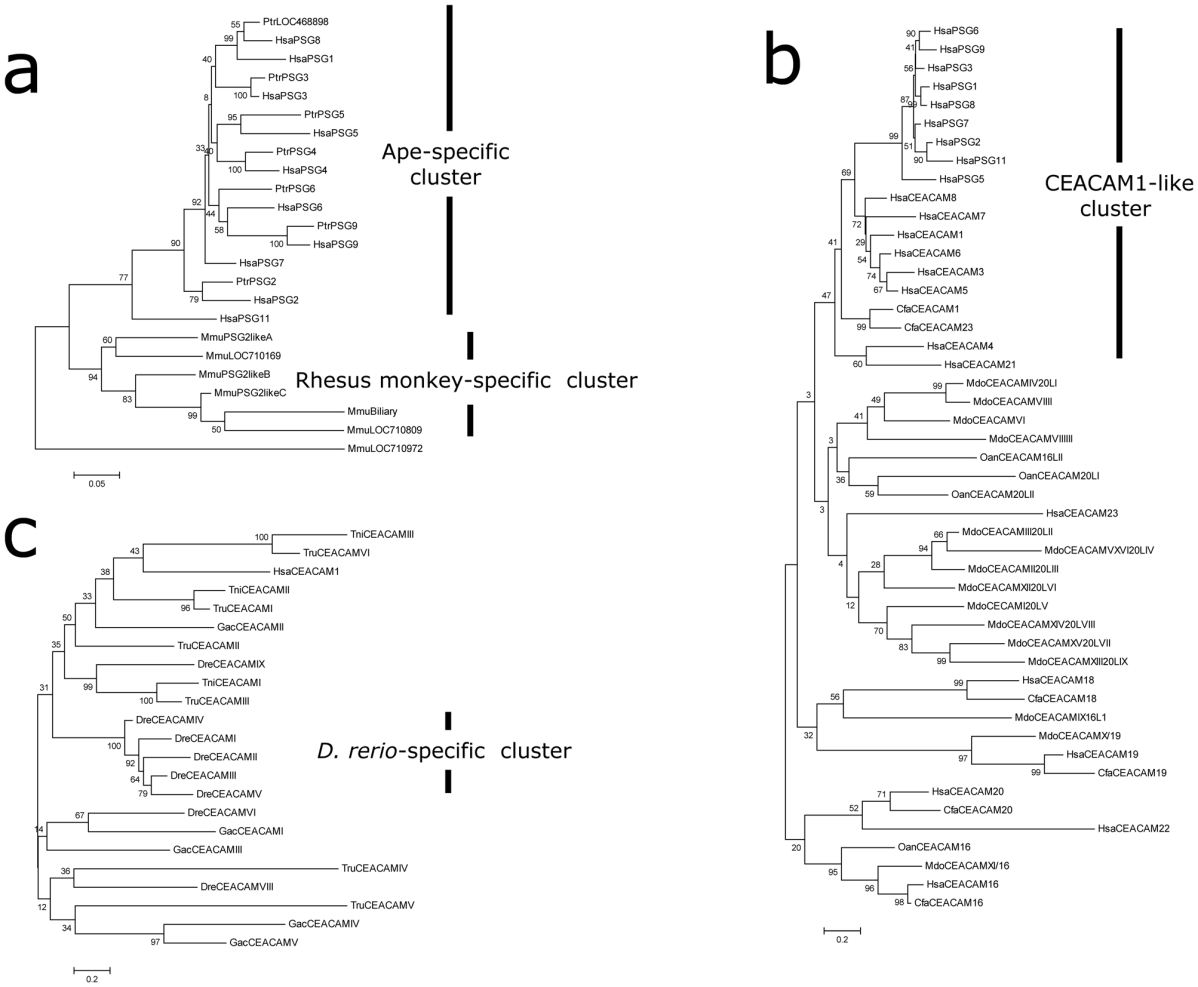
Analysis of CEACAM/PSG family gene evolution based on the Neighbor-Joining method. **a)** Phylogenetic tree of PSG subfamily genes from human, chimpanzee, and Rhesus monkey. A Rhesus monkey-specific cluster and an ape-specific cluster are indicated by vertical bars on the right. Potential pseudogenes, including human PSG10, chimpanzee LOC468901, and Rhesus monkey LOC709992, were excluded from the analysis. Human, Hsa; Chimpanzee, Ptr; Rhesus monkey, Mmu. **b)** Phylogenetic tree of 48 CEACAM family proteins from human, dog (*C. familiaris*), opossum (*M. domestica*), and platypus (*O. anatinus*). The analysis involved 48 protein sequences. There were a total of 2119 positions in the final dataset. The human CEACAM1-like cluster is indicated by a vertical bar on the right. The CEACAM16 and 20 homologs appear to diverge from other family members before the separation of eutherian, metatherian and prototherian mammals. Human, Hsa; dog, Cfa, opossum, Mdo; platypus, Oan. It is important to note that the bootstrap values for basal lineages in this tree are extremely low. The interpretation of this Neighbor-Joining tree has to be cautious**. c)** Phylogenetic tree of teleost CEACAM homologs. Twenty-three CEACAM proteins from *D. rerio*, *G. aculeatus*, *T. rubripes*, and *T. nigroviridis* were analyzed. A *D. rerio*-specific cluster is indicated by a vertical bar on the right. The robustness of the tree was assessed by 1,000 bootstrap replicates, and the percentage of replicates is shown next to the branches.

**Figure 3:**
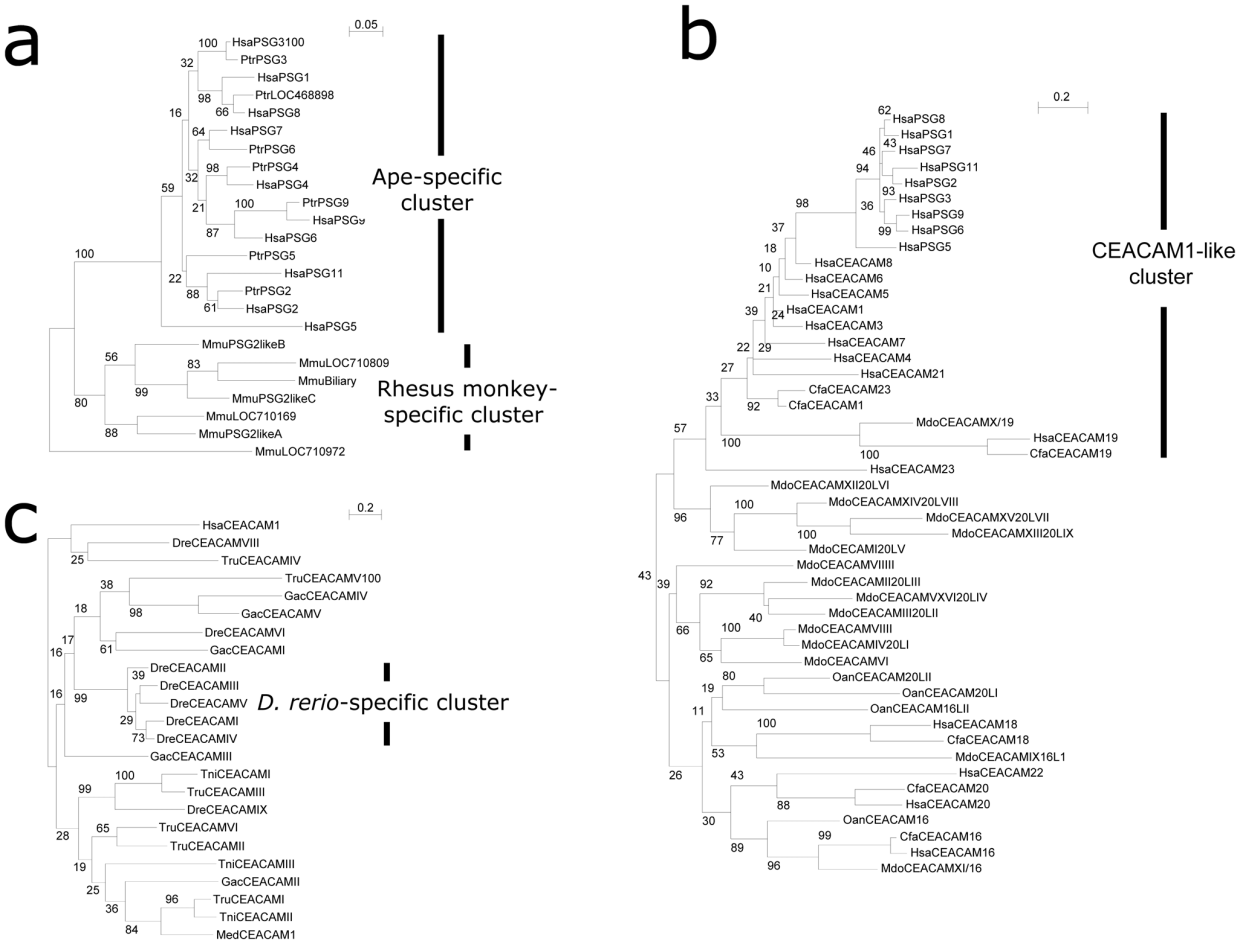
Analysis of CEACAM/PSG family gene evolution based on the Maximum Likelihood method. **a)** Phylogenetic tree of PSG subfamily genes from human, chimpanzee, and Rhesus monkey. A Rhesus monkey-specific cluster and an ape-specific cluster are indicated by vertical bars on the right. Potential pseudogenes, including human PSG10, chimpanzee LOC468901, and Rhesus monkey LOC709992, were excluded from the analysis. Human, Hsa; Chimpanzee, Ptr; Rhesus monkey, Mmu. **b)** Phylogenetic tree of 48 CEACAM family proteins from human, dog (*C. familiaris*), opossum (*M. domestica*), and platypus (*O. anatinus*). The analysis involved 48 protein sequences. The human CEACAM1-like cluster is indicated by a vertical bar on the right. Human, Hsa; dog, Cfa, opossum, Mdo; platypus, Oan. **c)** Phylogenetic tree of teleost CEACAM homologs. Twenty-three CEACAM proteins from *D. rerio*, *G. aculeatus*, *T. rubripes*, and *T. nigroviridis* were analyzed. A *D. rerio*-specific cluster is indicated by a vertical bar on the right.

In agreement with earlier studies, the CEACAM/PSG locus was found to be located near a cluster of marker genes, including *TGFB1*, *ATP1A3*, *ZNF574*, *PAFAH1B3*, *TMEM145*, *CNFN*, *LIPE*, *ETHE1*, *XRCC1*, *TOMM40*, *APOE*, and *SIGLEC8,* in a variety of eutherian genomes (Fig. 1, a and b) [20], [21]. In humans, chimpanzees, and Rhesus monkeys, this locus encodes 25, 18, and 24 CEACAM/PSG genes, respectively, in a 10-Mbp region on chromosome 19 (Fig. 1a, Table S1 in File S1). These primate CEACAM/PSG genes can be physically divided into two clusters. The cluster I contained the better characterized *CEACAM1,3–8* and *PSG* genes whereas the second cluster included the recently identified *CEACAM16, 18–20,* and *22–23* genes (Fig. 1a). Although the total number of CEACAM/PSG genes in Rhesus monkeys was close to that of humans, the majority of PSG-like genes in this primate did not show a 1∶1 orthologous relationship with those of apes. Instead, phylogenetic tree analysis using either Neighbor-Joining or Maximum Likelihood methods showed that most PSG-like genes in Rhesus monkeys clustered in a sub-branch in phylogenetic trees, suggesting that the expansion of PSG genes in Rhesus monkeys was independent of those in apes (Figs. 2a and 3a).

The number of CEACAM homologs in wet-nosed primates (Suborder *Strepsirrhin*; Lemuriforme, i.e., bushbaby and mouse lemur), which split from dry-nosed primates (Suborder *Haplorrhini*) ∼40 million years ago, was limited to 4–6 (Table S1 in File S1). Likewise, most non-primate mammals (e.g., cat, guinea pig, tree shrew, rabbit, squirrel, cow, hedgehog, microbat, shrew, elephant, small Madagascar hedgehog, and armadillo) contained 4–6 CEACAM homologs. There is an absence of PSG-like genes in these species, suggesting that PSG genes of dry-nosed primates were derived from lineage-specific duplications. Among non-primate eutherians investigated, rodents appeared to be the only group to have a large number of CEACAM homologs (16 in rats and 27 in mice) [20], [21], [42].

### Metatherian-specific Expansion of CEACAM Genes

Syntenic mapping showed that metatherian opossum encodes approximately three dozen CEACAM homologs with overall sequence similarities to *CEACAM1* and *5*. Among them, eleven homologs clustered with CEACAM/PSG locus marker genes TOMM40 and APOE on chromosome 4 (Fig. 1b; Table S2 in File S1). On the other hand, the prototherian platypus encoded only four CEACAM homologs (*OanCEACAM16, 16LI, 20LI,* and *20LII*)(Fig. 1b; Table S2 in File S1), and a pair of these homologs are positioned in tandem (Fig. 1b, Contig 2758). While CEACAM homologs from metatherian and prototherian mammals exhibit overall sequence similarity with eutherian counterparts, they have diverged extensively in domain structure and protein length as well as gene inventory, suggesting the absence of true orthologous relationship between these homologs and those of humans. Interestingly, although phylogenetic trees built with the Neighbor-Joining method showed little confidence on the relationship of most opossum and platypus homologs with those of eutherian homologs (Fig. 2b), phylogenetic trees built with the Maximum Likelihood method suggested that mammalian CEACAMs can be separated into two major branches: CEACAM16/18/20 and those clustered with the better characterized human CEACAM1-like members (Fig. 3b; Fig. S1 in File S1). In addition, the majority of metatherian homologs clustered in a sub-branch appeared to separate from those of human CEACAM1-like members.

### Divergent Evolution of CEACAM Family Genes in Basal Tetrapods and Teleosts

Syntenic mapping showed that CEACAM family genes are also present in genomes of basal tetrapods and teleosts (Fig. 1, b and c; Table S3 in File S1). However, no CEACAM homolog was identified in the genome of chicken, turkey (*M. gallopavo*), zebra finch (*T. guttata*), sea lamprey *(P. marinus*), and cephalochordate (amphioxus, *B. floridae*) and urochordates (tunicates, *C. intestinalis* and *C. savignyi*). In the clawed frog *X. tropicalis*, there are at least eight homologs, and three of them (XtrCEACAMI-III) were located close to marker genes including, LIPE, CNFN, TMEM145, PAFAH1B3, and ZNF574. In pufferfish, *T. rubripes* encoded at least six homologs (Fig. 1c; Table S3 in File S1), and three of these genes (scaffold 256, TruCEACAMI-III) were 5 Kbp from a PAFAH1B3 ortholog. Likewise, the homolog on scaffold 35 (TruCEACAMIV) was 20 Kbp from an XRCC1 ortholog (Fig. 1c). On the other hand, the closely related pufferfish *T. nigroviridis* contained only three homologs (TniCEACAM Ia, Ib, and II). TniCEACAM Ia and II were found near marker genes ZNF574 and ATP1A3, and PAFAH1B3, LIPE, TMEM145, and TOMM40a, respectively. By contrast, TniCEACAM Ib was located with a second TOMM40 ortholog (TOMM40b) and an ETHE1 ortholog. Based on the understanding that a WGD occurred before the divergence of teleosts and osteoglossomorphs more than 230–350 million years ago [30], [42], [43], TniCEACAM Ia and Ib as well as the pair of TOMM40 (a and b) genes likely represent WGD-derived co-orthologs.

Contrastingly, zebrafish and stickleback each contained at least ten CEACAM homologs (Fig. 1c; Table S3 in File S1). Most zebrafish homologs were found on two WGD-derived syntenic chromosome fragments that also contained co-orthologous ATP1A3 genes (ATP1A3a and 3b) as well as orthologs of ETHE1, ZNF574, PAFAH1B3, CNFN, LIPE, APOE, and TOMM40. In sticklebacks, at least five homologs on genome group XX (GacCEACAMI-V) were located close to marker genes CNFN, PAFAH1B3, XRCC1, and ETHE1. On the other hand, the medaka fish (*O. latipes*) contained only one CEACAM homolog on chromosome 16, which was surrounded by homologs of ATP1A3, PAFAH1B3, CNFN, LIPE, and TMEM145. Likewise, the West Indian Ocean coelacanth (*L. chalumnae*) contained at least one CEACAM homolog (DNA scaffold JH127065.1). Interestingly, a CEACAM homolog was also identified in a cartilaginous fish, little skate (*L. erinacea*; Class Chondrichthyes; FF598979.1, EE990618.1, and FF601895.1 in the EST database).

Furthermore, we found that hallmark sequences of the immunoglobulin superfamily (i.e., B cell receptors [BCRs], T cell receptors [TCRs], and FcRγ receptors), the cytoplasmic immunoreceptor tyrosine-based activation motif (ITAM) [44], [45], are present in select teleost homologs (Fig. S2 in File S1).

Taken together, these *in silico* analyses suggested that CEACAM-like gene found in basal taxonomy are orthologous to human CEACAM genes, but have undergone extensive local duplication or gene loss, resembling the repeated expansion of CEACAM/PSG genes in select therian mammals. In support of this observation, phylogenetic trees built with Neighbor-Joining and Maximum Likelihood methods indicated that five of the eight zebrafish CEACAMs on chromosome 16 (DreCEACAMI-V) form a sub-branch in which no homologs from other species are present, indicating that lineage-specific expansion could account for the large number of CEACAM homologs found in select fish species (Figs. 2c and 3c).

In addition, these data implied that, 1) CEACAM genes originated before the evolution of cartilaginous fish, 2) mammalian CEACAM/PSG genes evolve from one or two ancestral genes that were located adjacent to ATP1A3, ZNF574, PAFAH1B3, CNFN, LIPE, TOMM40, and APOE in the MRCA of teleost fish and tetrapods (Fig. S3 in File S1), and 3) CEACAM/PSG genes belong to an independent branch of the immunoglobulin superfamily that evolved in parallel with BCRs, TCRs, and FcRγ receptors early in vertebrates.

### Expression of CEACAM Homologs in Select Tissues of Platypus, Pufferfish and Zebrafish

**Figure 4:**
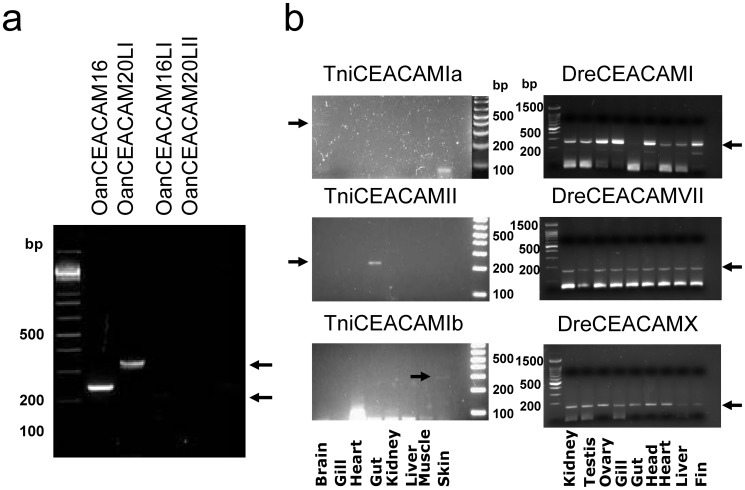
Analysis of CEACAM transcript expression in tissues of platypus, pufferfish *T. nigroviridis,* and zebrafish *D. rerio*. **a)** RT-PCR analysis of OanCEACAM16, 16LI, 20LI, and 20LII in the intestine of a platypus. Size markers are shown on the left. Specific PCR products for OanCEACAM16 and 20LI are indicated by arrows. **b)** RT-PCR detection of transcripts of DreCEACAMI, VII, and X in kidney, testis, ovary, gill, gut, head, heart, liver, and fin of *D. rerio* (right panel) as well as TniCEACAMI-III in brain, muscle, gut, kidney, heart, liver, gill, and skin of *T. nigroviridis* (left panel) using gene-specific primers (Table S4 in File S1). Expected size of PCR products for each gene is indicated by an arrow.

To verify that CEACAM homologs identified in lower taxonomy represent expressed genes, we analyzed the expression of select homologs in tissues of platypus, *T. nigroviridis,* and *D. rerio* using RT-PCR with gene-specific primers (Table S4 in File S1). Analyses of intestinal mRNA samples from a deceased platypus confirmed that at least two of the four platypus CEACAMs (OanCEACAM16 and 20LI) are constitutively expressed in the intestine, a tissue that exhibits pathological responses to overexpression of human CEACAMs in mice [46](Fig. 4a). Likewise, zebrafish homologs, including DreCEACAMI, VII, and X, were constitutively expressed in a variety of tissues (Fig. 4b, right panel). On the other hand, analyses of eight *T. nigroviridis* tissues showed that the expression of three CEACAMs in this species is tissue-specific. TniCREAMIa, Ib, and II are selectively expressed in the brain, skin, and gut, respectively (Fig. 4b, left panel).

### Human CEACAM/PSG Locus Exhibits Extensive Copy Number Variations

It has been hypothesized that the repeated expansion of CEACAM/PSG genes in primates and rodents could be associated with positive selection of select members [22], [23]. The hypothesis is supported by the finding that PSG genes in apes and Rhesus monkeys could be derived from independent expansion even though Rhesus monkey ancestors diverged from those of humans only 25 million years ago. This observation led us to further hypothesize that the selection shaping CEACAM/PSG gene repertoire in apes could be ongoing, and human CEACAM/PSG genes, as a group, could continue to diverge at a pace greater than that of other genes. To test this hypothesis, we investigated the extent of genetic variations (i.e., CNVs and SNPs) in human CEACAM/PSG genes, and whether the occurrence of CNVs and SNPs differs among human populations. In these analyses, richness and diversity of structural and nucleotide variations were used as proxies to determine the tendency for selection.

**Figure 5:**
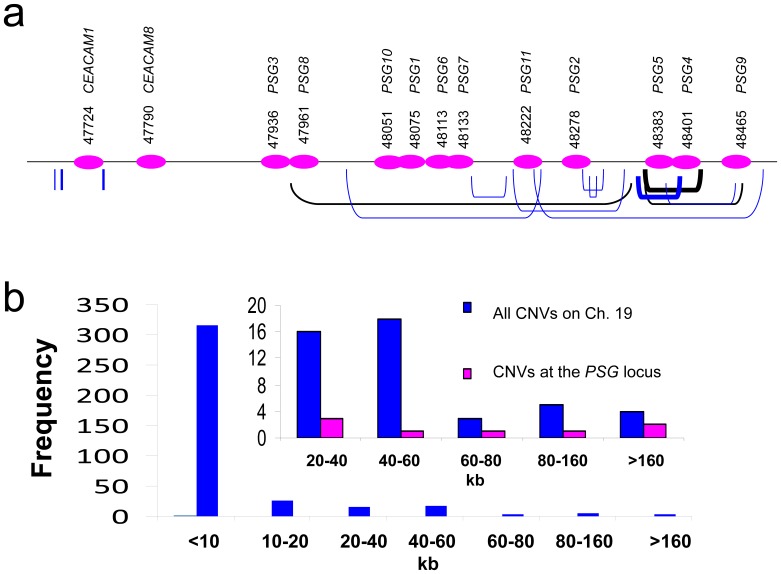
The human PSG locus exhibits frequent copy number variation (CNV). **a)** Schematic representation of CNVs found at the human PSG locus on chromosome 19 (47,600–48,500 kb) based on studies using high-density probes and DNA sequencing [51], [52], [53]. CNVs that were identified in CEU (U.S. residents with northern and western European ancestry, N = 20) and YRI (Yoruba from Ibadan in African, N = 20) populations are indicated by blue brackets under the chromosome. CNVs that were identified in Asian populations (Chinese, Japanese, and Koreans; N = 30) are indicated by black brackets. CNVs that have a frequency higher than 50% are indicated by bold brackets. **b)** The size distribution of 387 unique chromosome-19 CNVs that have a length greater than 500 bp. The figure in the inset shows the distribution of long fragment CNVs (46 in total have a length >20 kb) found on chromosome 19 and those at the PSG locus.

Analysis of structural variations based on earlier array studies using low-density probes showed the presence of various types of gain and/or loss of genomic fragment encompassing CEACAM/PSG genes [47], [48], [49], [50]. These CNVs range from a few kb to as large as 0.8 Mb in a small fraction of individuals (Fig. S4 in File S1). Consistently, recent data based on high-density probe comparative genome hybridization analysis and/or genome sequencing indicated that CNVs at the CEACAM/PSG locus are pervasive, and most of these CNVs are results of large segmental duplications (Fig. 5a) [51], [52], [53].

Whereas there are at least 387 unique CNVs that are larger than 500 bp (range from 0.5 to 379 kb) on chromosome 19, 14 of them affected the CEACAM/PSG locus. Of the 46 CNVs that encompassed a >20-kb region, a disproportionate 17.4% (8 out of 46 CNVs) of them were located at the CEACAM/PSG locus (Fig. 5b). Three of these CNVs actually represented the longest gains or losses of chromosome segment on chromosome 19, and all these CNVs affected multiple PSG genes: CNVR3824.1 (379 kb, encompassing *PSG1, 2, 6, 7, 10,* and *11*), CNVR7658.2 (238 kb, including *PSG1, 6, 7, 10,* and *11*), and CNVR7658.5 (252 kb, including *PSG2, 4, 5,* and *9*)(Table S5 in File S1). Among CNVs, those have a frequency higher than 1% are distinguished as copy number polymorphisms. Based on this criterion, almost all CEACAM/PSG CNVs represented common copy number polymorphisms (>3% frequency) that have been retained in recent human history. Furthermore, we estimated that the copy number of PSG genes could range from 12 copies in individuals who are homozygous for gene loss at CNVR7658.2 to 30 copies in individuals with homozygous gene gains at CNVR7658.5, instead of 22 copies found in most individuals.

In addition, CNVs at the CEACAM/PSG locus exhibited high population differentiation (Table S5 in File S1). For example, CNVR7655.1 and CNVR7656.1 were found in 5% of the CEU (U.S. residents with northern and western European ancestry) populations, but not in the YRI (Yoruba from Ibadan in African) populations. On the other hand, CNVR7657.1 was unique to YRI populations (15%). By contrast, the CNVR3825.1 that encompassed *PSG4* is specific to ∼80% of East Asians (ASN, pooled samples of Chinese, Japanese, and Koreans).

Given that gain or loss of a whole set of genes are rare events in human genomes, these observations indicated that gene duplication/retention and loss occurred frequently at the CEACAM/PSG locus, and these events could be under strong selection after the separation of major human populations in the last 40–80 thousand years.

### SNPs in Human CEACAM/PSG Genes Exhibit High Population Differentiation

Because the redundancy of CEACAM/PSG genes could distort common methods used to detect population differentiation of SNPs (including the so-called outlier approaches, in which candidate loci were identified in the extreme tails of empirical distributions, and neutrality tests), we used two alternative approaches to test whether there is an enhanced richness and diversity of SNPs in human CEACAM/PSG genes. First, we compared the density of synonymous and nonsynonymous gene-region SNPs in CEACAM/PSG genes to those of other genes on chromosome 19. Although the density of synonymous SNPs within CEACAM/PSG genes was similar to the average of other genes, the density of nonsynonymous SNPs in CEACAM/PSG genes was significantly higher than that of the rest of genes on chromosome 19 (Table 1, *p*<0.01). Consistently, whereas 40% of CEACAM/PSG genes contained at least one nonsynonymous SNP, only 8.3% of the 1,363 other genes on chromosome 19 contained nonsynonymous SNPs (Table 2). These data suggested that CEACAM/PSGs are enriched with genetic diversity in the coding region.

**Table 1:** CEACAM/PSG family genes have a high density of nonsynonymous SNPs.

Groups	Average number of nonsynonymous SNPs	Average number of synonymous SNPs
Other genes on Ch. 19	3.8±0.3	3.6±0.2
CEACAM/PSG genes	8.3±1.6*	4.2±0.9

* significantly different from the chromosome average (P<0.01).

**Table 2:** A large fraction of human CEACAM/PSG genes contain nonsynonymous SNPs.

Groups	Total gene number	Genes with nonsynonymous SNPs	Percentage with nonsynonymous SNPs
**Annotated genes on Ch. 19**	1363	113	8.29
**CEACAM/PSG genes**	25	10	40*

* significantly different from the chromosome average (Chi-squared test, P<0.01).

**Figure 6:**
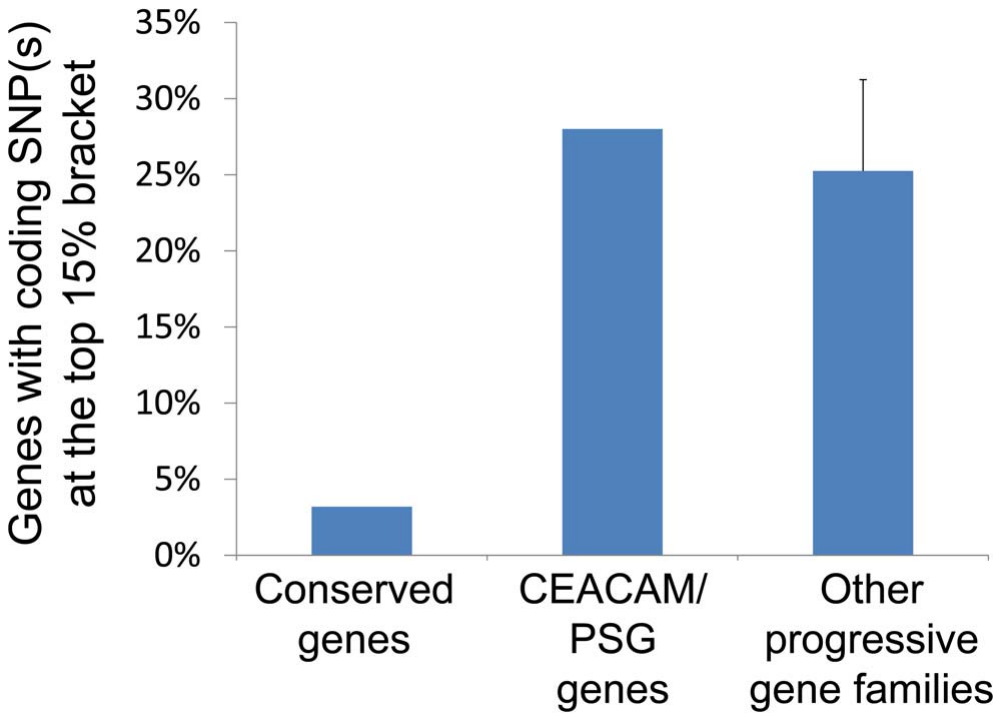
The CEACAM/PSG and three other progressive gene families (sialic acid binding Ig-like lectin, leukocyte immunoglobulin-like receptor, and olfactory receptor) have a higher percentage of genes containing a nonsynonymous SNP with F_ST_ scores in the top 15% bracket as compared to that of the rest of genes (conserved genes) on chromosome 19. Progressive gene families are those encoded secreted ligands or cell surface receptors, and expanded multiple times during primate evolution.

Second, we analyzed whether high-frequency nonsynonymous SNPs (i.e., has an average frequency >10%) in CEACAM/PSG genes exhibit population differentiation in the International HapMap II populations using the empirical distribution of the F_ST_ statistic, which has been widely used to detect alleles that were selected after human populations left Africa ∼60 thousand years ago. In support of our hypothesis, we found that only 3.2% (N = 43) and 2.6% (N = 34) of a total of 1327 non-CEACAM/PSG genes contained at least one coding SNP with F_ST_ scores in the top 15% and 10% bracket of all SNPs, respectively. On the other hand, seven and three out of 25 CEACAM/PSG genes (28% and 12%) had at least one coding SNP with F_ST_ scores in the top 15% and 10% bracket, respectively. In addition, we found that eight out of these 25 genes contained gene region SNPs with F_ST_ scores in the top 5% bracket (Table S6 in File S1). In addition to CEACAM/PSG genes, chromosome 19 encodes several gene families that exhibit functional and evolutional characteristics similar to CEACAM/PSGs. These progressive gene families includes sialic acid binding Ig-like lectin, leukocyte immunoglobulinlike receptor, and olfactory receptor gene families [29], [31], [42], [43], [54], [55]. They encode secreted ligands or cell surface receptors, and the family size expanded multiple times during the evolution of primates. Interestingly, 25% and 19.4% of genes in these three families contained at least one nonsynonymous SNP with F_ST_ scores in the top 15% and 10% bracket, respectively (Table 3). Therefore, the ratio of genes containing coding SNP(s) with a high F_ST_ score in CEACAM/PSG and these three progressive gene families is categorically higher than other genes (conserved genes) on chromosome 19 (Fig. 6, Table 3).

**Table 3:** Percentage of genes with nonsynonymous SNP(s) that exhibit a high F_ST_ score (in the top 15% or 10% bracket).

Category	Conserved gene families	CEACAM/PSG	Other gene families with a high number of new members in primates			
			Olfactory receptor	Sialic acid binding Ig-like lectin	Leukocyte immunoglobulin-like receptor	Total
**Total number of genes**	1327	25	17	8	11	36
**With SNP at top 15% fraction**	43	7	5	3	1	9
**Ratio**	3.2%	28.0%	29.4%	37.5%	9.1%	25.0%
**With SNP at top 10% fraction**	34	3	4	2	1	7
**Ratio**	2.6%	12.0%	23.5%	25.0%	9.1%	19.4%

The analysis included four progressive genes families (CEACAM/PSG, sialic acid binding Ig-like lectin, leukocyte immunoglobulin-like receptor, and olfactory receptor) and the rest of genes (conserved genes) on chromosome 19.

These high F_ST_ CEACAM/PSG SNPs included several *CEACAM1* polymorphisms (F_ST_ scores in the top 1% bracket: rs8111171, rs8110904, and rs8111468) that were repeatedly identified as targets of positive selection in human populations [56], [57]. The ancestral allele at rs8110904 was found in 99% of CEU populations whereas the derived allele was found in 100% of ASN populations (Table S6 in File S1). On the other hand, the derived allele of nonsynonymous SNPs in *CEACAM8* (rs8103051) and *PSG11* (rs10414166) were unique to YRI and CEU populations, respectively. The SNP rs12610545 in *CEACAM18* was unique to Eurasians (Table S6 in File S1). Likewise, analysis based on the CEPH-HGDP project (Centre d’Etude du Polymorphisme Humain-Human Genome Diversity Panel; 944 unrelated individuals from 52 populations) [58], [59], [60] showed that nonsynonymous SNPs in *PSG4, CEACAM1, CEACAM18,* and *CEACAM21* are among those with the highest population differentiation in world populations (Fig. S5 in File S1).

In addition, analysis of linkage disequilibrium (LD) at the CEACAM/PSG locus among HapMap II populations indicated that *CEACAM7-5-6*, *PSG11-2-5-4,* and *CEACAM21-4* regions exhibit long-range LD patterns. Importantly, LD patterns in these regions are population specific. In the *CEACAM7-5-6* region, extensive LD was obvious only in CEU and ASN, but not YRI populations (Fig. S6a in File S1). At the *PSG11-2-5-4* region, a unique pattern of LD was only shared by CEU and YRI populations (Fig. S6b in File S1). On the other hand, extensive LD at the *CEACAM21-4* region was only found in ASN populations (Fig. S6c in File S1).

## Discussion

Syntenic mapping of chordate genomes showed that CEACAM orthologs are present in lower taxonomy, and the number of CEACAM homologs ranges from zero in amphioxus, tunicates, sea lamprey and chicken to more than three dozen in the opossum. The radical divergence of gene inventory occurred even among species under the same family, perhaps reflecting the extensive birth-and-death of CEACAM/PSG genes in response to environmental selection. Interestingly, CEACAM/PSG genes appeared to evolve in a similar manner in humans. The CECAM/PSG locus contained a higher density of nonsynonymous SNPs compared to other genes on the same chromosome. In addition, these genetic variations exhibited high population differentiation in geographically separated human populations. Similar to studies of SNPs, we found that structural variations are common at the PSG locus, and these copy number polymorphisms exhibit population differentiation. Together with earlier studies on the dN/dS ratio in primate and rodent species [22], [23], these results indicated that CEACAM/PSG genes had a more dynamic evolutionary history than previously thought.

In the last decade, significant progress has been made in our understanding of CEACAM/PSG genes from species-specific searches to the elaborate analysis of positive and negative selection among species [22], [23]. However, due to the extensive divergence in CEACAM/PSG sequences, it has been difficult to extrapolate animal studies to humans and vice versa, thereby limiting our understanding of the molecular and biochemical nature of CEACAM/PSG signaling. Here, syntenic mapping has clarified the relationship of various CEACAM-like genes identified in noneutherian mammals and basal taxonomy. In addition, our analysis implied that the characteristic CEACAM-signaling pathway that involves the recruitment of downstream molecules such as phosphatidylinositol 3-kinase, SHP1 phosphatase, and RAC-GTPase by ITAM or ITIM motifs could have evolved in the MRCA of teleosts and tetrapods, but not in urocordates, cephalochordates or sea lamprey [7], [61]. Furthermore, our analysis indicated that CEACAM/PSG gene family represents an independent branch of the immunoglobulin superfamily. Earlier analyses of adaptive immunity have shown that jawed and jawless vertebrates developed antigen receptors independently, and the hallmark of adaptive immunity of vertebrates, including major histocompatibility complex molecules and T/B-cell receptors originated in jawed vertebrates [42], [54]. Thus, the immunoglobulin variable and constant domain-containing CEACAM family genes have evolved in parallel with BCRs, TCRs, and FcRγ receptors in jawed vertebrates.

Changes in selection pressures over time could leave diverse footprints of selection in genes. In general, the methods for detecting the action of selection vary with timescales. On the longest timescales, selection is evidenced by differences in gene inventory and gene functions. At shorter timescales, selection is indicated by an excess of nonsynonymous changes as compared to synonymous substitutions. At time scales <80 thousand years, positively selected SNPs in humans could be detected by an excess of derived alleles in LD because there is no sufficient time for the genetic diversity to be restored by mutation and drift [56], [62]. However, none of these studies alone could provide a comprehensive view of how divergent the evolution of a gene family was. This is particularly true for gene families that contain redundant members. In addition, varied arrangements of functional domains among paralogs, such as those found in CEACAM/PSG genes, could add to the challenge of analysis of gene evolution [56], [62]. In the present study, we broadened the study of CEACAM/PSG gene evolution in two directions: the identification of CEACAM homologs in basal taxonomy (long timescale) and the investigation of genetic diversity in humans during a short timescale (i.e., after the Out-of-Africa human migration). On the first front, we found that CEACAM genes evolved in a radical manner in various lineages. In an extreme instance, these genes were completely lost (i.e., avian species). On the second front, we took two complementary approaches– local variations in density of coding SNPs/CNVs and population-based assessment of genetic variations–to assess the extent of divergence. In these analyses, genes on chromosome 19, rather than the genome-wide samples, were selected to account for local substitution rate variation. In these analyses, we found CEACAM/PSG genes contain high density of nonsynonymous SNPs and CNVs, and many of these genetic variations exhibit high population differentiation, indicating extensive gene–environmental interactions at the CEACAM/PSG locus. In addition, these data suggested that the selection force shaping the expanded CEACAM/PSG gene inventory in Old World primates is ongoing in humans. Whereas the underlying mechanism remains to be investigated, the evolutionary course of these genes could be a result of combinations of factors.

First, the high frequency of genetic variations at the CEACAM/PSG locus could be a result of relaxed constraint. Recent studies have revealed that an estimated 5–10% of the vertebrate gene repertoire consist of ―progressive (vs. conserved) gene families which mainly function in immunity, reproduction, and chemosensory [25], [63], [64], [65], and that ligands and cell surface receptors could be preferentially retained after gene duplications, perhaps due to low constraints imposed by fewer interacting protein partners compared to intracellular polypeptides that form complexes with many partners in two-way communication [66], [67]. Because CEACAMs and PSGs mainly function as cell surface receptor or secreted ligand, their evolution could be similar to that of a variety of progressive genes, including olfactory receptors, male gamete-associated reproductive genes, KIRs, and other immune-associated gene families, which experienced frequent birth-and-death of duplicated genes in response to environmental pressures.

Second, the frequent occurrence of CNVs at the PSG locus could be partly attributed to the presence of repetitive sequences. It is well accepted that frequent gene gain and gene loss in progressive genes could be the result of unequal crossover of genes with repeated domains, which can promote rearrangements through their own repeatability [68], [69]. Similarly, recent studies have shown that CNV mutation rates in duplication-rich regions could be significantly higher than SNPs due to their propensity to undergo nonallelic homologous recombination, thereby making CNV to be recurrent at specific loci [70]. Physiologically, the redundancy of CEACAM/PSG genes could be selected for the provision of a robustness against null phenotypes in individuals [71], [72]. In support of this hypothesis, it has been shown that transgenic mice deficient for *Ceacam1*, *9*, or *10* exhibit minimal or no aberrant phenotypes [3], [73], [74]. In addition, the enhanced redundancy could, theoretically, further reduce the constraint and add new opportunities for genetic drift or selection to operate on new alleles, thereby allowing the accumulation of nonsynonymous SNPs in duplicated PSG genes. However, it is important to note that the presence of repeated sequences alone could not account for the selection of high genetic variations at the CEACAM/PSG locus. Recent studies of CNV hotspots in humans have identified several duplication-rich regions on chromosome 19, which contain clusters of zinc finger proteins (ZNFs, with a total of 204 members on chromosome 19) [75]. However, these ZNF cluster loci did not exhibit appreciable increases of genetic variations as compared to other genes on chromosome 19. For example, the ratio of ZNF family genes that have a high F_ST_ nonsynonymous SNP (5.4%) is similar to that of other genes on the same chromosome (3.2%).

Third, genetic variations at the CEACAM/PSG locus could be associated with functional elements that were selected by the environment. Recent analyses of SNPs showed that human genomes contain hundreds of loci that exhibit signatures of positive selection (i.e., gene–environmental interactions) [56], [76], and a number of these genetic variants are associated with the evolution of a variety of common traits (e.g., appearance, physiological adaptations) and pathologies in humans [76], [77], [78], [79]. Likewise, CNVs have been associated with a number of human phenotypes and diseases [80], [81]. For example, amylase CNVs were highly associated starch contents of the food among human populations [82], whereas, CNVs at the CCL3L-CCL4L locus were associated with the susceptibility to auto-immune disorders, HIV-1 infection, and asthma [83], [84], [85]. While PSG physiology could be dosage insensitive (PSG copy number could range from 12 to 30 in normal individuals), the observation that CEACAM/PSG variations are comparable to those of genes under rapid adaptive evolution in primates [86], [87] suggested that CEACAM/PSG variation-associated changes in functional characteristics or gene expression could help determine survival and reproductive success of the carrier.

Fourth, CEACAM/PSG genes, particularly PSGs, could be selected to provide a strict immunocompatibility between the mother and the infant, or to broaden pathogen resistance during pregnancy. Given that CEACAM/PSGs could act through homophilic and heterophilic interactions [7], [9], the pressure to select new variants could be self-enforced if the carrier of a new immuno-modulation CEACAM/PSG variant favors fetus with the new variant as a result of immunocompatibility. By the same token, this mechanism could also contribute to the high population differentiation of select CEACAM/PSG variants. Therefore, our finding could represent an important vehicle for exploring novel genotype–phenotype relationships in PSG-mediated fetal–maternal interactions. In addition, our study raised the possibility that genetic variations at the PSG locus could be involved in the manifestation of reduced fertility and various pregnancy complications in select individuals. In support of our speculation, a recent report indicated that there could be a nominal relationship between PSG11 gene copy variation and the occurrence of preeclampsia [88].

In a nutshell there are at least four factors, including (1) relaxed selection associated with cell surface molecules, (2) gene redundancy and functional robustness, (3) continual selection of functional elements, and (4) the maintenance of immunocompatibility, that could have contributed to the survival advantage and divergence of CEACAM/PSG genes.

## Supporting Information

File S1 **The supporting information file contains a total of six supplemental figures (Figures S1–S6) and six supplemental tables (Tables S1–S6).** Figure S1. Alignment of CEACAM homologs. Figure S2. Identification of the immunoreceptor tyrosine-based activation motif (ITAM) in the cytoplasmic domain of zebrafish CEACAMs. Figure S3. Schematic representation of a putative evolutionary trajectory of CEACAM genes in teleosts and tetrapods. Figure S4. Schematic representation of CNVs found at the CEACAM/PSG locus based on studies using low-density probes (International HapMap Project webserver). Figure S5. Distribution of allele frequencies of select nonsynonymous SNPs of *PSG4, CEACAM1, CEACAM18,* and *CEACAM21* in HGDP-CEPH world populations. Figure S6. SNPs in CEACAM7-5-6, PSG11-2-5-4, and CEACAM21-4 regions are highly linked in select human populations. Table S1. Inventory of CEACAM family genes in human, chimpanzee, Rhesus monkey, bushbaby, and mouse lemur. Table S2. Inventory of CEACAM family genes in the opossum and the platypus. Table S3. Inventory of CEACAM family genes in nonmammalian vertebrates including, *X. tropicalis, D. rerio, G. aculeatus, T. nigroviridis,* and *T. rubripes*. Table S4. PCR primers for the amplification of select CEACAM transcripts in tissues of the platypus, *T. nigroviridis,* and *D. rerio.* Table S5. PSG-locus CNVs that were identified based on high-density probes [51], [52], [53]. Table S6. A large proportion of human CEACAM/PSG genes contain SNPs with high population differentiation.
(PDF)
