# Supplementary material for: Widespread Divergence of the CEACAM/PSG Genes in Vertebrates and Humans Suggests Sensitivity to Selection
Source: PLoS One. 2013 Apr 16;8(4):e61701. doi: 10.1371/journal.pone.0061701 (PMC3628338; doi:10.1371/journal.pone.0061701)
Supplement: File S1 — The supporting information file contains a total of six supplemental figures (Figures S1–S6) and six supplemental tables (Tables S1–S6). Figure S1. Alignment of CEACAM homologs. Figure S2. Identification of the immunoreceptor tyrosine-based activation motif (ITAM) in the cytoplasmic domain of zebrafish CEACAMs. Figure S3. Schematic representation of a putative evolutionary trajectory of CEACAM genes in teleosts and tetrapods. Figure S4. Schematic representation of CNVs found at the CEACAM/PSG locus based on studies using low-density probes (International HapMap Project webserver). Figure S5. Distribution of allele frequencies of select nonsynonymous SNPs of PSG4, CEACAM1, CEACAM18, and CEACAM21 in HGDP-CEPH world populations. Figure S6. SNPs in CEACAM7-5-6, PSG11-2-5-4, and CEACAM21-4 regions are highly linked in select human populations. Table S1. Inventory of CEACAM family genes in human, chimpanzee, Rhesus monkey, bushbaby, and mouse lemur. Table S2. Inventory of CEACAM family genes in the opossum and the platypus. Table S3. Inventory of CEACAM family genes in nonmammalian vertebrates including, X. tropicalis, D. rerio, G. aculeatus, T. nigroviridis, and T. rubripes. Table S4. PCR primers for the amplification of select CEACAM transcripts in tissues of the platypus, T. nigroviridis, and D. rerio. Table S5. PSG-locus CNVs that were identified based on high-density probes [51], [52], [53]. Table S6. A large proportion of human CEACAM/PSG genes contain SNPs with high population differentiation. (PDF) [file pone.0061701.s001.pdf]

The Content of Supporting Information File S1:

Figure S1. Alignment of CEACAM homologs. a) Alignment of CEACAM16 orthologs from human, dog, opossum, and platypus. The two immunoglobulin variable (IgV)-like regions at the N-terminus and C-terminus are indicated by blue bars, whereas the two immunoglobulin constant (IgC)-like regions are indicated by red and yellow bars below the sequences, respectively. **b)** Alignment of CEACAM19 orthologs from human, dog, and opossum. The single IgV-like region at the N-terminus is indicated by blue bars below the sequences. **c)** Alignment of select zebrafish CEACAM homologs on chromosome 16 and human CEACAM1. The single IgV-like region at the N-terminus is indicated by blue bars below sequences whereas the two IgC-like regions are indicated by red and yellow bars, respectively. The transmembrane and cytoplasmic domain sequences of DreCEACAMI were not included in the alignment. Residues that are identical in the majority of aligned sequences are highlighted by a dark background.

Figure S2. Identification of the immunoreceptor tyrosine-based activation motif (ITAM) in the cytoplasmic domain of zebrafish CEACAMs. The consensus ITAM domain is shown together with the ITAM sequences of human CEACAM1 and 3 (upper panel) [11,22,45]. The conserved YXX/I/L/V/M sequences and the conserved acidic residue preceding the tyrosine residue are shown in red letters. Conserved ITAM sequences in the cytoplasmic region of DreCEACAMI and IV are indicated by red letters, and the critical tyrosine residues are underlined (lower panel). The transmembrane (TM) sequences preceding the cytoplasmic region are underlined.

Figure S3. Schematic representation of a putative evolutionary trajectory of CEACAM genes in teleosts and tetrapods. Based on syntenic mapping and phylogenetic analyses, we deduced that CEACAM family genes originated with a couple of ancestral genes in the MRCA of teleosts and tetrapods. These ancestral genes were located in close proximity to marker genes, including ATP1A3, ZNF574, PAFAH1B3, CNFN, LIPE, TOMM40, and APOE. In teleosts, CEACAM homologs could be derived from WGD and tandem duplications. In tetrapods, only chimpanzees contained PSGs orthologus to human counterparts. CEACAM and PSG homologs are indicated by red and green diamond-shaped symbols on the chromatid, respectively.

Figure S4. Schematic representation of CNVs found at the CEACAM/PSG locus based on studies using low-density probes (International HapMap Project webserver) [47,48,49,50]. Gains or losses of genomic fragments are indicated by horizontal bars under the chromosome.

Figure S5. Distribution of allele frequencies of select nonsynonymous SNPs of *PSG4, CEACAM1, CEACAM18,* and *CEACAM21* in HGDP-CEPH world populations. The graphic presentation was generated using the HGDP selection browser (http://hgdp.uchicago.edu/cgi-bin/gbrowse/HGDP/). Pie charts represent the proportion of each genotype by geographic region. The ancestral and derived alleles are indicated by blue pie and yellow pie, respectively.

Figure S6. SNPs in CEACAM7-5-6, PSG11-2-5-4, and CEACAM21-4 regions are highly linked in select human populations. a) Plots of linkage disequilibrium (LD) between each pair of genotyped SNPs in a 140-kb region surrounding the CEACAM7-5-6 region (chr19:46,840 kb

– 46,980 kb) in HapMap II populations (Release 24). The LD was analyzed using Haploview 4.2 [41]. Genes are indicated by lines and blocks above the LD plots. In CEU and ASN, all three genes are covered by large LD blocks. In YRI, LD was minimal at the same genomic region. The color scheme was based on r2 values. Red areas represent regions with a high degree of LD and a high likelihood of odds (LOD) (D' =1, LOD scores >2). Blue areas represent regions with low LOD (D' =1, LOD<2). **b)** LD plots in a 200-kb region surrounding the PSG11-2-5-4 region (chr19:48,200 kb – 48,400 kb). At the *PSG11* and *PSG4* regions, a high degree of LD is most obvious in CEU and ASN, respectively. **c)** LD plots in a 100-kb region surrounding the CEACAM21-4 region (chr19:46,730 kb – 46,830 kb). At this locus, extensive LD was found in the 5' and the gene regions of *CEACAM21* in ASN populations.

Table S1. Inventory of CEACAM family genes in human, chimpanzee, Rhesus monkey, bushbaby, and mouse lemur.

Table S2. Inventory of CEACAM family genes in the opossum and the platypus.

Table S3. Inventory of CEACAM family genes in nonmammalian vertebrates including, *X. tropicalis, D. rerio, G. aculeatus*, *T. nigroviridis,* and *T. rubripes*.

Table S4. PCR primers for the amplification of select CEACAM transcripts in tissues of the platypus, *T. nigroviridis,* and *D. rerio.*

Table S5. PSG-locus CNVs that were identified based on high-density probes [51,52,53].

Table S6. A large proportion of human CEACAM/PSG genes contain SNPs with high population differentiation.

Fig. S1.

A.

```
HsaCEACAM16      --MSDLLSIYSAPVVVSTVLHMLQIRKLRLGRVHKLPRVLEVGNSGTMSRAYTFFFLRWS 58
CfaCEACAM16      ------------------------------------------------------------
MdoCEACAMXI/16   ----MSDGHTNALSRPRPKRSQDQQRSPQGLHKIPGTSTRLPGLRLGVTNGTALGQLGLP 56
OanCEACAM16      -----------MEFQALSSARISSGKTLNSPFAERLTAAPPGGGAARMAAVASPSGCGVP 49

HsaCEACAM16      FMALTG-YSWLLLSATFLN-----VGAEISITLEPAQPSEGDNVTLVVHGLSGELLAYSW 112
CfaCEACAM16      -MAVTG-CSWLLLSATFLS-----VGAEISITPEPAQPAEGDNVTLAVHGLLGELLAYNW  53
MdoCEACAMXI/16   PTGAPGPWKGLLLTAVFLMAWIPPAPALLNILVIPEPPAQGDNVTLLVRGLPGELLAYNW 116
OanCEACAM16      PARWGRTWTGLLLAASVLTAWLPPAPAQLTVTPIPPNPLEGWDVTLSVSGAPGGLLLYNW 109
                         . ***:* .*      . * :.:   *  * :* :*** * *  * ** *.*

HsaCEACAM16      YAGPTLSVSYLVASYIVSTGDETPGPAHTGREAVRPDGSLDIQGILPRHSGTYILQTFNR 172
CfaCEACAM16      YAGPTLSLTYLVASYIVSTGDETPGPAHTGREAVRPDGSLDIQGVLPRHSGTYILQTLNR 113
MdoCEACAMXI/16   YRGTNLNQAHLILSYIISTADETPGPAYTGREAVRPDGSLNLRDVVPEDSGSYILQTLNK 176
OanCEACAM16      YRGASLSLTQMILSYINATEIQTPGAAHSGREAVHPNGSLLIQRVTLNDSGSYLLQSINP 169
                 * *..*. : :: *** :*  :***.*::*****:*:*** :: :  ..**:*:**::*

HsaCEACAM16      QLQTEVGYGHVQVHEILAQPTVLANSTALVERRDTLRLMCSSPSPTAEVRWFFNGGALPV 232
CfaCEACAM16      QLQTEVGYGHLQVYEILAQPVVMANNTALVERRDTLRLMCSSPSP-AEVRWFFNGEALPI 172
MdoCEACAMXI/16   QFQTDIAYGHLLVHEILSPPLLLANGTELVERRDTIHLLCSTPST-GDVRWFFNGEALPI 235
OanCEACAM16      QFQTEIAFGFLRVYERFSKPRVIANGTNVVEYRDSVELTCVTSHM-ADIRWYFNHQLIPG 228
                 *:**::.:*.: *:* :: * ::**.* :** **::.* * :.   .::**:**   :*

HsaCEACAM16      ALRLGLSPDGRVLARHGIRREEAGAYQCEVWNPVSVSRSEPINLTVYFGPERVAILQDST 292
CfaCEACAM16      AIRLGQSPDGRVLTRHGIRREEAGAYQCEVWNPVSVSRSEPINLTVYFGPERVAILQDST 232
MdoCEACAMXI/16   GSRLGLTPDNRILVRHNVRREEAGAYQCEISNPVSVSRSDPVNLTVYYGPDRVTIIQESA 295
OanCEACAM16      SS--------RVLMRPGVRREEAGVYQCEVWSLLSANRSDPVQLTVNYGPDHVTITQESA 280
                 .         *:* * .:******.****: . :*..**:*::*** :**::*:* *:*:

HsaCEACAM16      TRTGCTIKVDFNTSLTLWCVSRSCPEPEYVWTFNGQALKNGQDHLNISSMTAAQEGTYTC 352
CfaCEACAM16      ARTGCTIKLDFNTSLTLWCVSQSCPEPEYVWAFNGRALKNGRDHLNISSMTAAQEGTYTC 292
MdoCEACAMXI/16   SQTGCTVKVELDASLTLWCVTRSCPDPEYIWSFNGRTHESTTSFVNITGMTKDKEGTYTC 355
OanCEACAM16      LRKGCTIEAELNSTLTLWCVTESCPEPQYEWTLNGTSRGRPQDSLLIGAMSWEHQGAYTC 340
                  :.***:: :::::******:.***:*:* *::** :     . : * .*:  ::*:***

HsaCEACAM16      IAKNTKTLLSGSASVVVKLSAAAVATMIVPVPTKPTEGQDVTLTVQGYPKDLLVYAWYRG 412
CfaCEACAM16      IAKNPKTLLSGSASVVVKLTAAAVAMTIVPVPTKPMEGQDVTLTVQGYPKDLLVYAWYRG 352
MdoCEACAMXI/16   IAKNPITELTGSASVLVKMLVATSAMTIVPVPSKPTEGKDVILSVQGYPKDLLVYAWYRG 415
```

```
OanCEACAM16          IAKNNKTQLSASATVFLTVTVMASSFMIVPIPARQVEGQDVTLSVQGCPRDLLVYAWYRG 400
                     ****  * *:.**:*.:.: . : :  ***:*::  **:** *:*** *:**********

HsaCEACAM16          PASEPNRLLSQLPSGTWIAGPAHTGREVGFPNCSLLVQKLNLTDTGRYTLKTVTVQGKTE 472
CfaCEACAM16          PASEPNRLLSQLPSGNWIAGPAHTGREVGFANCSLLVQKLNLTDAGRYMLKTVTLQGKTE 412
MdoCEACAM16          TASEPNRLLSQLPSGNWIAGPAHTGREMGFANCSLLIQKLNLTDTGRYTLKTVTLQGKTE 475
OanCEACAM16          TPDEPNRLVSQLPLGNWIAGPAHSGRETGFPNCSLHIQHLNASDSGRYTLKTVTLQGKTE 460
                     ...*****:**** *.*******:*** **.**** :*:** :*:*** *****:*****

HsaCEACAM16          TLEVELQVAPLG------------------------------------------------ 484
CfaCEACAM16          TLEVELQVAPLE------------------------------------------------ 424
MdoCEACAM16          TLDVQLQVSIPYLITITIIIFIIIIITGVSTSACCLCYPHFSRTFAFPKHFPANTTPQHS 535
OanCEACAM16          MLEIELRVL--------------------------------------------------- 469
                      *:::*:*
```

b.

```
HsaCEACAM19     MEIPMGTQGCFSKSLLLSASILVLWMLQGSQAALYIQKIPEQPQKNQDLLLSVQGVPDTF  60
CfaCEACAM19     MEIPEWAQHYFSKGLLLSASILALWIPQGSWAALRIQKIPEYPQKDQDLLLSVQGIPGNF  60
MdoCEACAMX/19   -MVLLHGGGWVWKEFMFTASLLAWWTVQQAWAGLLIEKIPETPQEGQDVLLTVHGVPAAI  59
                  :       . * ::::**:*. *  * : *.* *:**** **:.**:**:*:*:*  :

HsaCEACAM19     QDFNWYLGEETYGGTRLFTYIPGIQRPQRDGSAMGQRDIVGFPNGSMLLRRAQPTDSGTY 120
CfaCEACAM19     QDFNWYLGEETNGGTMLFTYFPDLQWPQRDGSAMGQHDIVGFPNGSMMLHRVQPTDSGTY 120
MdoCEACAMX/19   KDFNWYQGEEVDGSTMIFSYFPGLPRPQRNGNALQGRNIIGFPNGSFLLRHVQLTDSGIY 119
                :***** ***. *.* :*:*:*.:  ***:*.*:  ::*:******::*::.* **** *

HsaCEACAM19     QVAITINSEWTMKAKTEVQVAEKNKELPSTHLPTNAGILAATIIGSLAAGALLIS-CIAY 179
CfaCEACAM19     QVAVNINPAWIMRAKTEVQVAEKHKELPIIHLPVSAGIMAAIIIGSLSAGSLFIC-CIAH 179
MdoCEACAMX/19   QVGITFNPSWIMRAKTELKVIGEWH----SSSAVNSGTLVAIVLGCVGLGALIVGGGLAY 175
                **.:.:*. * *:****::*  : :       ...:* :.* ::*.:. *:*::   :*:

HsaCEACAM19     LLVTRNWRGQSHRLPAPRGQGSLSILCSAVSPVPSVTPSTWMATTEKPELG----PAHDA 235
CfaCEACAM19     LLLTGGWRGQSHR----------------------------MTPTEKPEAR----PNLNA 207
MdoCEACAMX/19   LMVSRGWRIRSPG-----------------------------NITVKPEPGRDGCQKHRA 206
                *::: .** :*                                 * ***          *

HsaCEACAM19     GDNNIYEVMPSPVLLVSPISDTRSINPARPLPTPPHLQAEPENHQYQQDLLNPDPAPYCQ 295
CfaCEACAM19     GDQHIYEVMPSPTHLVSPLGGTASMNNTMPLPQQ---QPEPENHPY-QDLLNPDPDPYCQ 263
MdoCEACAMX/19   DGSNIYEVIHSPGILIAPTSVTGIINPNLTPDSP---GICPGVAHCLHVYHEAGPKRWGS 263
                ...:****: **  *::* . *  :*   .          *      :   :..*  : .

HsaCEACAM19     LVPTS 300
CfaCEACAM19     LTPTH 268
MdoCEACAMX/19   -----
```

C.

```
DreCEACAMI          -----------MMVSHGLFWTLWMVACFEQCLGQDLQFPELNN-GAVGGSVKFTPNNPPS  48
DreCEACAMII         -----------MMASNSLYWILWMVTCFGEYLGQDLQFPGQTY-GAIGGSVMFTPENLGS  48
DreCEACAMIII        -----------MTVPRGLYWTLWVVACFGQSFNQDLQFPELTN-GAVGESVKFTPDNIPS  48
DreCEACAMV          ------------MVSHGLLWTLWMLACFGQSFGEDFQFPELTN-GAVGGSVKFTPNNLPS  47
hCEACAM1            MGHLSAPLHRVRVPWQGLLLTASLLTFWNPPTTAQLTTESMPFNVAEGKEVLLLVHNLPQ  60
                                   ..*     ::: :      ..         * *  * .   *

DreCEACAMI          TAIN-QFTWQFE-------TILILTGKPDSPTVSSEYQDRVFLDKNTLALELWNLTFRDS 100
DreCEACAMII         IGIDVQITWNFG-------ATNILTGKPESPTIVPAYVDRVSFDQNTLSLELRNLKMEDS 101
DreCEACAMIII        EILS--VSWHFG-------DIFILNGNPDSPLIFPAYEDKVSFDKNTLALELWDLKLEDS  99
DreCEACAMV          EIDT--VTWQFG-------QTFILSGNPDTASIVSAYKNRSSFDKNTLALELWNLKLEDA  98
hCEACAM1            QLFG--YSWYKGERVDGNRQIVGYAIGTQQATPGPANSGRETIYPN-ASLLIQNVTQNDT 117
                           :*                  .: .   .   .:  :  *  :* : ::. .*:

DreCEACAMI          GSYNLSVNTVNGDEIREQTSLQVYEIISDVTLTGPQE--TLIEDESSANITSKGSG-NIT 157
DreCEACAMII         GSYILTVTPVSGDQLRGETTLQLFENITNVRLTGPEE--TLIEDESFANITSEGSG-TIT 158
DreCEACAMIII        GSYSLTVITSRGNSHKGETSLQVFEKINYVAVTGPHETEALIEGESSANFTSKGNG-NVT 158
DreCEACAMV          GPYSLTVIS-NGNQHRGETSLQVFETIYHVTVTGPQT-EALIEGESSANFTSEGSGGTIT 156
hCEACAM1            GFYTLQVIKSDLVNEEATGQFHVYPELPKPSISSNNS--NPVEDKDAVAFTCEPETQDTT 175
                    * * * *      . .    ::::  :    ::. .     :*.:. . :*.: .    *

DreCEACAMI          SVQWMKDNSPLSSSSRIIFSSDNRSVYISPVERSDTGELQCTYSNPVSSETAK-HTLIIN 216
DreCEACAMII         SVQWMKDNSPLSSSSRIIFSSDNRSVSISPVERSDTGEYQCTYSNPVSSETAK-LTLIIN 217
DreCEACAMIII        SVQWMKDNSPLSSSSRIIFSSDNRSVSISPVQRSDTGEYQCTYSNPVSSETAK-LILIIN 217
DreCEACAMV          SVQWMKDNSPLSPNSRIIFSSDNRSVSISPVQRSDTGEYLCIYSNPVSSASNS-QPLIIN 215
hCEACAM1            YLWWINNQS-LPVSPRLQLSNGNRTLTLLSVTRNDTGPYECEIQNPVSANRSDPVTLNVT 234
                     :*::::* *. ..*: :*..**:: : .* *.***   *  .****:   .   * :.

DreCEACAMI          YGPDGVSIEGPNV-VDLGVQIFLSCSANSEPSASFSWTFNGSDTGVTTDKYTIDKTDFNN 275
DreCEACAMII         YGPEGVSIKGPDE-MDLGVQVFLSCFANSEPSASFSWTLNGSDTGVTTDKYTIDKTDFTD 276
DreCEACAMIII        YGPEGVSIKGPDM-VDLGVQVFLSCSANSEPSASFSWTFNGSDTGVTKDALTIDKTDFTD 276
DreCEACAMV          YGPDDVSITGPDV-VDLEVQVSLSCSAKSEPSASFNWTFNGSDTGVNTDNFTIEKTDFTD 274
hCEACAM1            YGPDTPTISPSDTYYRPGANLSLSCYAASNPPAQYSWLING-TFQQSTQELFIPNITVNN 293
                    ***:  :*  .:      .:: *** * *:*.*.:.* :**     ..:   * :  ..:

DreCEACAMI          SGEYICTAFNRVTNRRELKTHALLVQAGGGGLSGGAIAGIVIGVLV-------------- 321
DreCEACAMII         SGEYICVAQPPALQLSVKEAPHVNAKEDAMLCVECALTLGGHGSPWLR------------ 324
DreCEACAMIII        SGEYICIKCSSCTQVKK------------------------------------------- 293
DreCEACAMV          SGEYISQRGQDAELNYADISHFQKNSGERVVLGDVNEPRTEYAEVRHGSKLRAPPPPY-  332
hCEACAM1            SGSYTCHANNSVTGCNRTTVKTIIVTE--------------------------------  320
                    **.* .                   .
```

Consensus ITAM ***D/E***XX***YXXL/I***(X)$_{7-12}$***YXXL/I/V/M***

hCEACAM3 ***YEEL***LKHDTNI***YCRM***DH

hCEACAM1 ***E***VT***YSTL***NFEAQQPTQPTSASPSLTATE-LL***YSEV***

DreCEACAMI TM and cytoplasmic domains

AGIVIGVLVAVSGICGLIVYLTKTNKIP

KLKSEAQRSEAPAKSKEKT**D**LT**YVNI**PGHQMTRGGETAQGKGGDSAI

YMNMKTDPNSKEDE**YVNV**KHGVPSSQR*

DreCEACAMIV TM and cytoplasmic domains

AGAIAGIVIGVLVAVGGICGLIVYFT

KTNKIPTQRLQHKGQASGAAQRGQEL**D**LH**YEEI**TDSQRARENFNLEN

ISEAQT**YENV**A**Y**KSD**YENL**KHGVLKQPQASEP*

Fig. S2

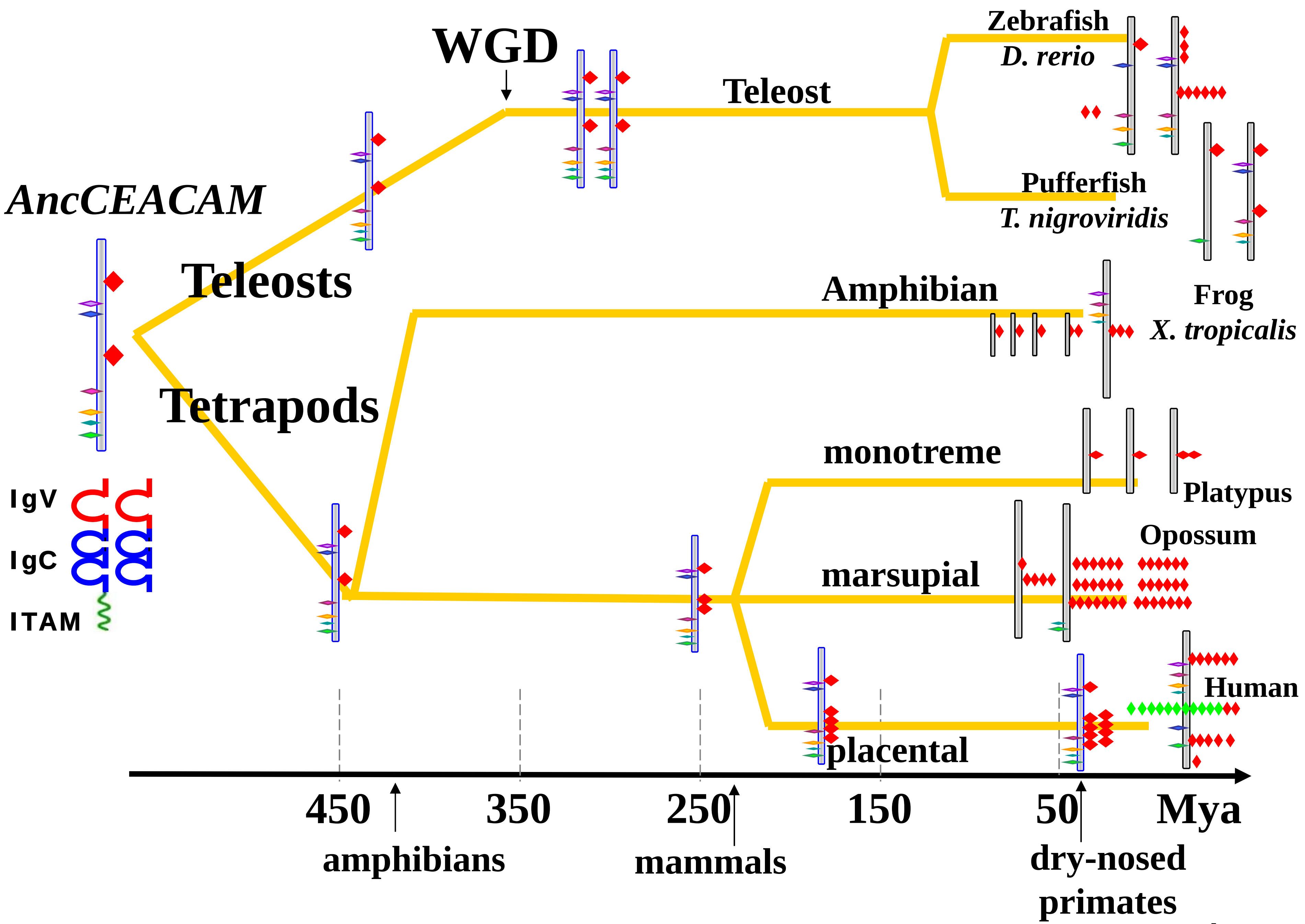

AncCEACAM
Teleosts
Tetrapods
WGD
Teleost
Zebrafish
D. rerio
Pufferfish
T. nigroviridis
Amphibian
Frog
X. tropicalis
monotreme
Platypus
Opossum
marsupial
Human
placental
IgV
IgC
ITAM
450
350
250
150
50
Mya
amphibians
mammals
dry-nosed
primates

Fig. S3

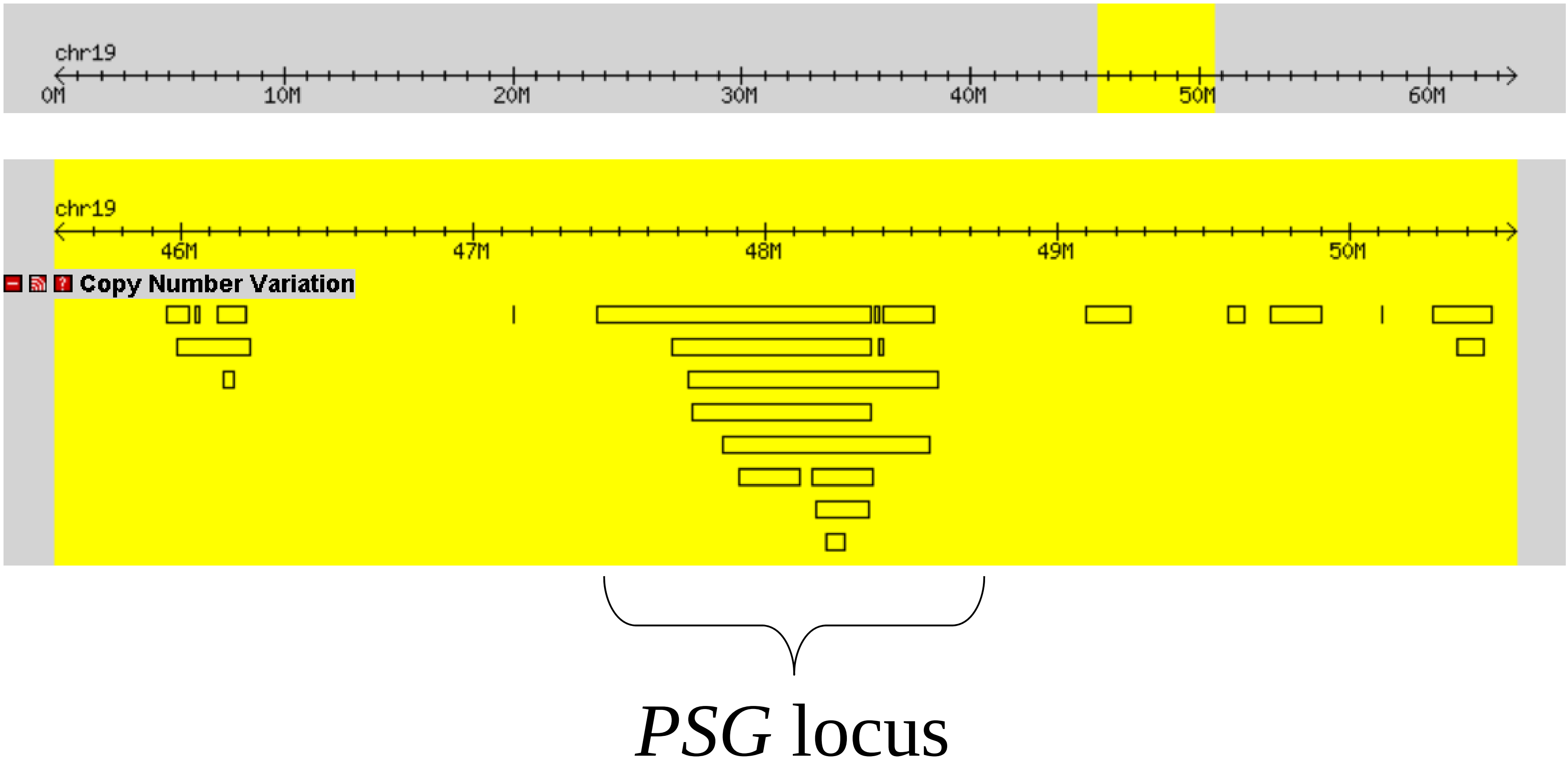
chr19
0M
10M
20M
30M
40M
50M
60M
chr19
46M
47M
48M
49M
50M
Copy Number Variation
PSG locus

Fig. S4

PSG4 (rs3859474)

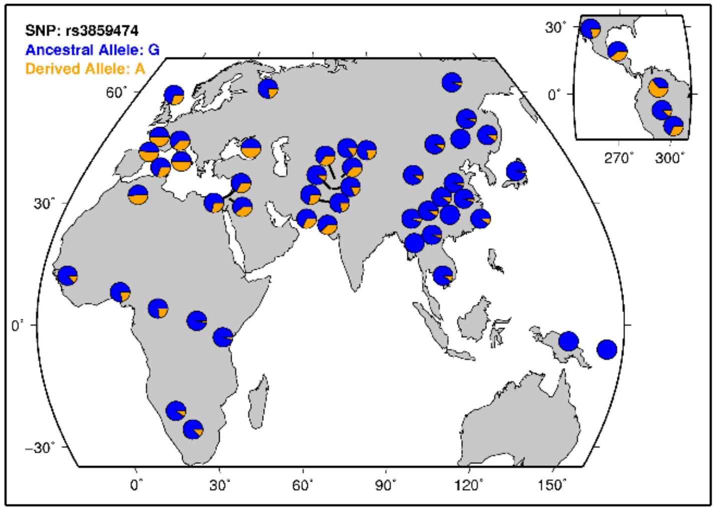

CEACAM1 (rs8110904)

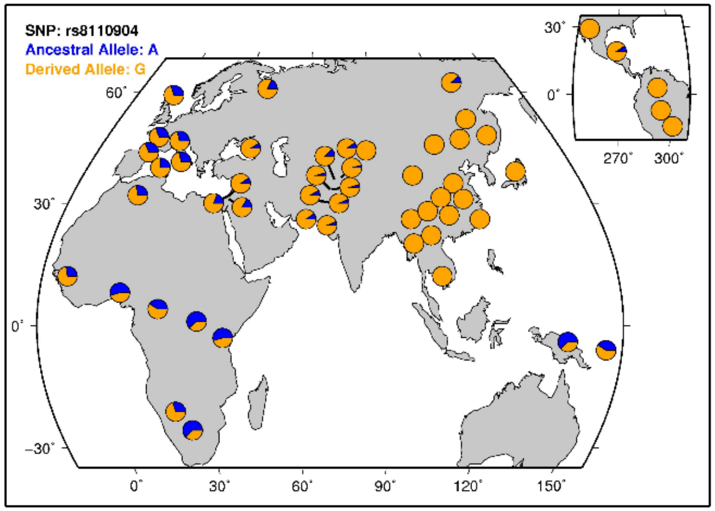

CEACAM18 (rs12610545)

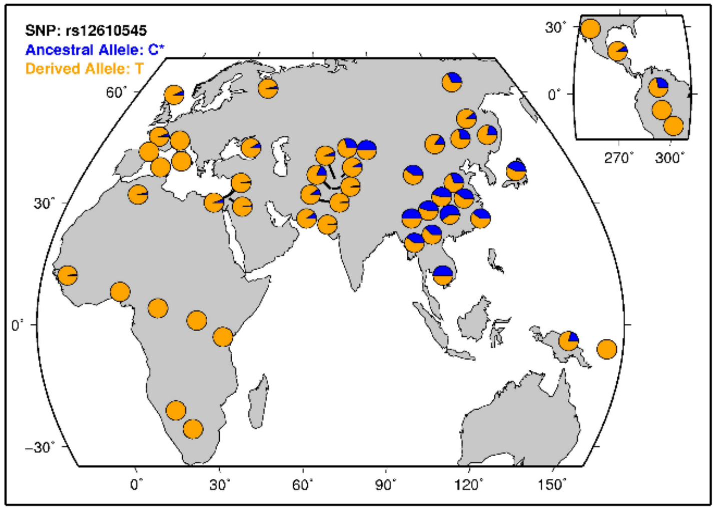

CEACAM21 (rs714106)

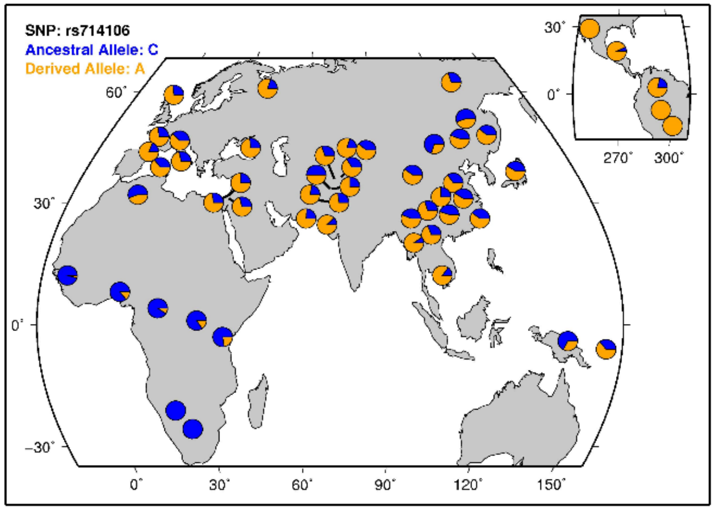

Fig. S5

a

CEACAM7-CEACAM5-CEACAM6

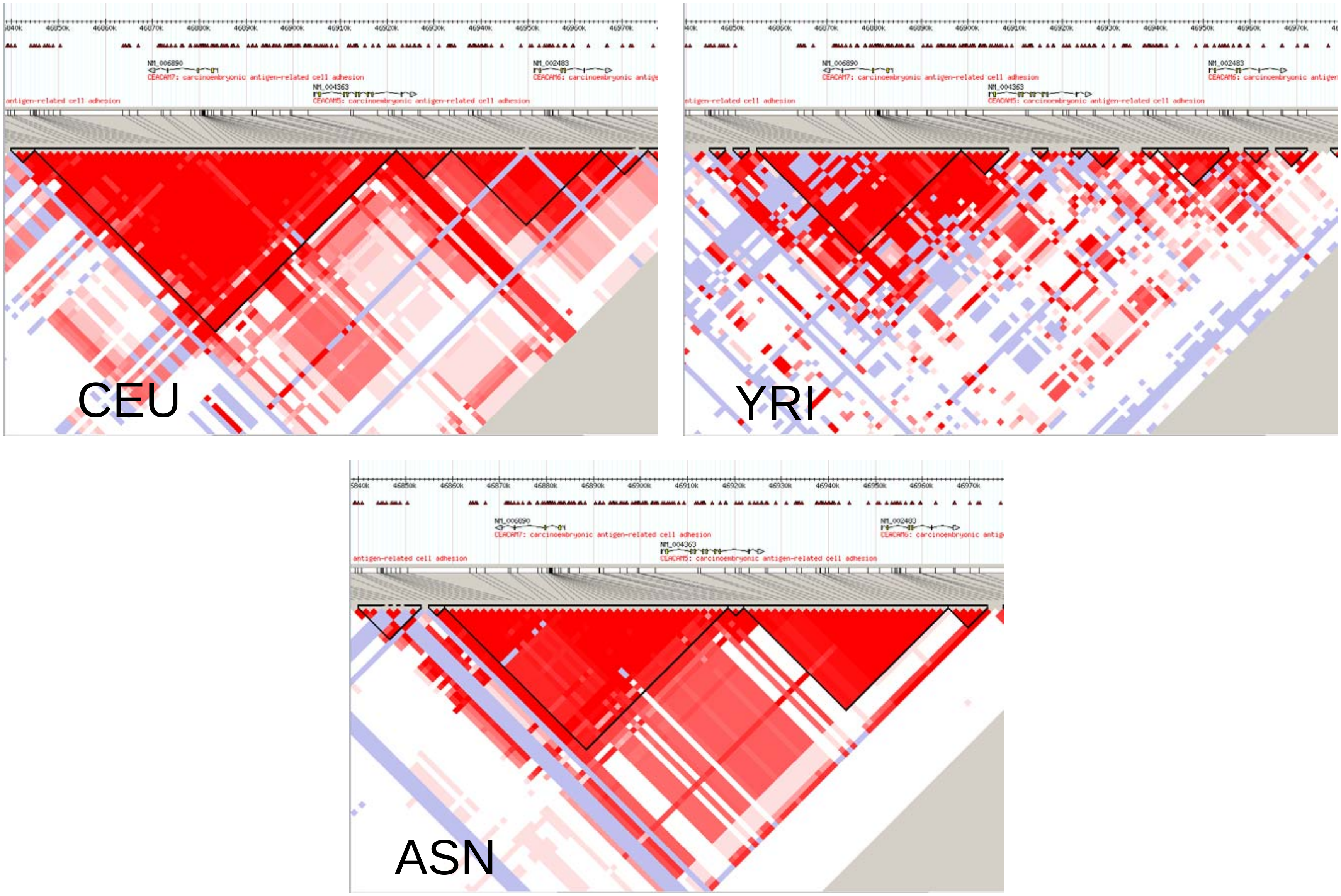

Fig. S6

b

PSG11-PSG2-PSG5-PSG4

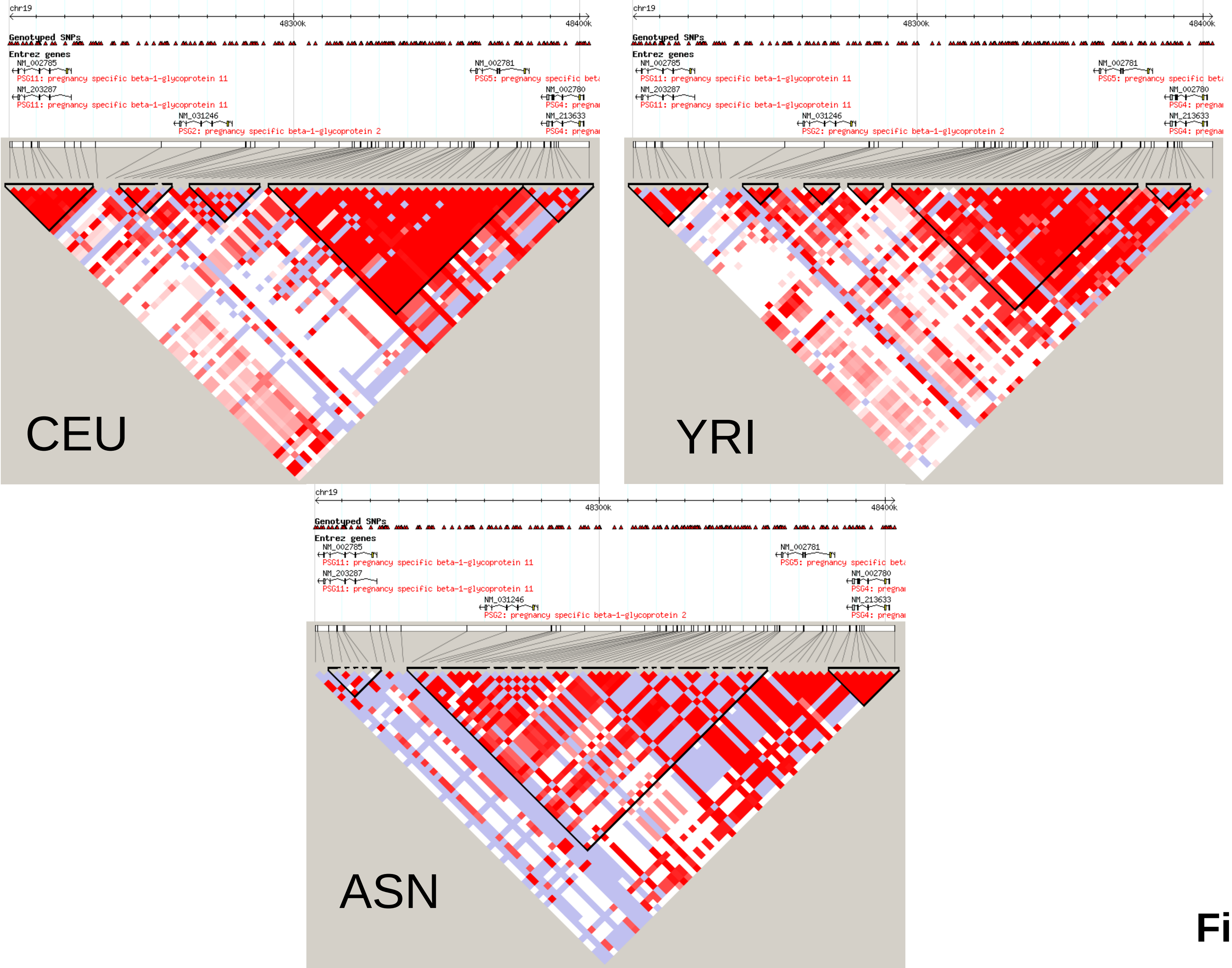

Fig. S6

C

CEACAM21-CEACAM4

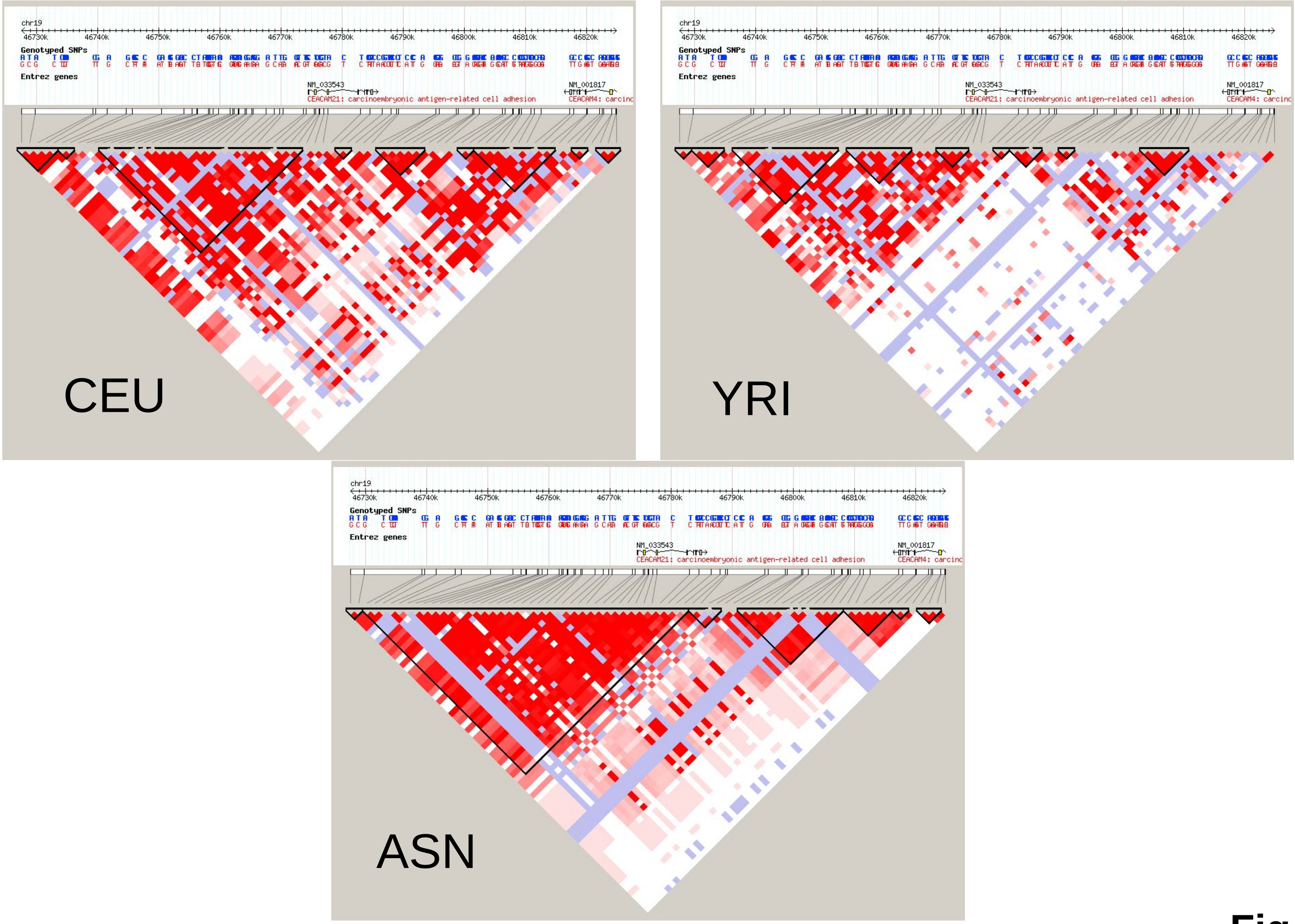

Fig. S6

Table S1. Inventory of CEACAM family genes in human, chimpanzee, Rhesus monkey, bushbaby, and mouse lemur.

Human			
**Marker genes**	**position (Kbp)**		**Accession No.**
	**Start**	**End**	
TGFB1	46551	46528	BAF84596
ATP1A3	47190	47162	EAW57089
ZNF574	47266	47277	NP_073589
PAFAH1B3	47498	47493	EAW57126
TMEM145	47509	47521	EAW57130
CNFN	47586	47583	EAW57134
LIPE	47623	47597	BAF85171
ETHE1	48723	48702	Q0JUX8
XRCC1	48771	48739	EAW57203
TOMM40	50086	50098	ABM87186
APOE	50100	50104	ABM86685
SIGLEC8	56653	56646	BAF83101
**CEACAM homologs**			
hCEACAM21	46774	46785	Q3KPI0
hCEACAM4	46825	46817	EAW57056
hCEACAM7	46883	46869	EAW57058
hCEACAM5	46904	46925	ABM87752
hCEACAM6	46951	46967	EAW57061
hCEACAM3	46992	47007	Q3KPH9
hCEACAM1	47724	47703	Q3KRG8
hCEACAM8	47790	47776	ABM86087
hPSG3	47936	47916	ABW03894
hPSG8	47961	47950	AAI42629
hPSG10	48033	48051	Q15228
hPSG1	48075	48063	Q0JSJ3
hPSG6	48113	48098	ABM87387
hPSG7	48133	48120	NP_002774
hPSG11	48222	48203	NP_002776
hPSG2	48278	48260	NP_112536
hPSG5	48383	48363	EAW57173
hPSG4	48401	48388	NP_998798.1
hPSG9	48465	48449	NP_002775
hCEACAM20	49725	49697	NP_001096070
hCEACAM22/FLJ41856	49751	49732	XP_946155.1
hCEACAM23/LOC147710	49808	49831	XP_946160.1
hCEACAM19	49866	49879	BAF84277

hCEACAM16	49894	49905	AAI56856
hCEACAM18	56671	56678	NP_001073874
Chimpanzee			
**Marker genes**	**position (Kbp)**		**Accession No.**
	**Start**	**End**	
TGFB1	46919	46894	XP_512687
ATP1A3	47473	47443	XR_023337
ZNF574	47554	47560	Pseudo
PAFAH1B3	47789	47782	XP_001153954
TMEM145	47801	47812	XP_001142679
CNFN	47874	47870	XP_001154605
LIPE	47912	47885	XR_024357
ETHE1	49079	49058	XP_512716
XRCC1	49133	49097	XP_001156934
APOE	50503	50507	NP_001009007
SIGLEC8	57116	57096	XP_512856
**CEACAM homologs**			
CEACAM4	47105	47083	XP_512688.2
CEACAM7	47156	47142	XP_524278.1
CEACAM5	47178	47277	XR_023262
LOC456078	48014	47992	XR_025573
CEACAM8	48080	48065	XP_512705
PSG3	48229	48207	XP_001143535
LOC468898-PSG8	48253	48240	XP_524284
PSG5	48363	48345	XP_512707
PSG6	48448	48376	XP_512707
PSG2	48604	48525	XP_512709.
PSG4	48747	48705	XP_001144127
LOC468901	48762	48753	NW_001228230
PSG9	48810	48794	XP_001144286
LOC469085	48835	48817	XP_524470
LOC468913	50114	50097	NW_001228236
LOC456114	50257	50269	XR_024745
LOC456116	50286	50298	XR_024752
LOC469102	57123	57130	XP_001156934
Rhesus monkey			
**Marker genes**	**position (Kbp)**		**Gene ID**
	**Start**	**End**	
TGFB1	47746	47723	GeneID: 574135
ATP1A3	48303	48272	GeneID: 706513

ZNF574	48383	48395	GeneID: 706729
PAFAH1B3	48623	48617	GeneID: 707570
TMEM145	48636	48646	GeneID: 709268
LIPE	48752	48725	GeneID: 707997
XRCC1	50034	49999	GeneID: 711457
TOMM40	51318	51331	GeneID: 713297
APOE	51333	51341	GeneID: 714623
SIGLEC6	57616	57600	GeneID: 719684
**CEACAM homologs**			
LOC705453-CEACAM4	47973	47962	GeneID: 705453
LOC705572-CEACAM7	48019	48008	GeneID: 705572
CEACAM5	48036	48049	GeneID: 705689
LOC707824	48083	48138	GeneID: 707824
LOC708200	48852	48829	GeneID: 708200
LOC708398-CEACAM8	48918	489039	GeneID: 708398
LOC709707	49059	48988	GeneID: 709707
LOC709992	49204	49201	GeneID: 709992
PSG2-LIKEa	49259	49239	GeneID: 710079
LOC710169	49319	49295	GeneID: 710169
Biliary glycoprotein 1	49497	49654	GeneID: 710532
PSG2-LIKEb	49607	49580	GeneID: 709407
PSG2-LIKEc	49654	49635	GeneID: 710710
LOC710809	49708	49686	GeneID: 710809
LOC710972	49760	49724	GeneID: 710972
LOC711053	49800	49788	GeneID: 711053
LOC711132	49829	49808	GeneID: 711132
LOC714015-CEACAM20	50912	50891	GeneID: 714015
LOC714065	50922	50915	GeneID: 714065
LOC714249-CEACAM22	51037	51063	GeneID: 714249
LOC714411-CEACAM19	51106	51118	GeneID: 714411
LOC714515-CEACAM16	51135	51146	GeneID: 714515
LOC719898	57438	57427	GeneID: 719898
LOC719939-CEACAM18	57549	57563	GeneID: 719939

Bushbaby	
**Marker genes**	**position**
ATP1A3	Genescaffold GeneScaffold_1073 at location 25,969-55,762.
LIPE	GeneScaffold_1074 at location 182,407-199,360.
ZNF574	GeneScaffold_1095 at location 50,500-59,648.
TMEM145	GeneScaffold_3950 at location 4,206-15,171.
CNFN	GeneScaffold_2804: 41.98k
ETHE1	GeneScaffold_1096: 39.03k

XRCC1	Genescaffold GeneScaffold_1096: 206.21k
APOE	Genescaffold_86060 at location 6,253-7,597.
TOMM40	GeneScaffold_2025 at location 49,105-73,048.
**CEACAM homologs**	
ENSOGAG00000012575	GeneScaffold_1072 at location 135-8,183.
ENSOGAG00000002909	GeneScaffold_529 at location 70,975-94,165.
ENSOGAG00000000088	Genescaffold_86083 at location 41,236-45,879.
ENSOGAG00000006840	Genescaffold GeneScaffold_3930: 154.58k
Mouse Lemur	
**Marker genes**	**position**
ATP1A3	Genescaffold_12218 at location 13,156-21,970.
LIPE	GeneScaffold_967 at location 369,130-392,998.
TGFB1	GeneScaffold_942 at location 66,233-85,704.
PAFAH1B3	GeneScaffold_967 at location 281,481-283,361.
TMEM145	GeneScaffold_967 at location 290,954-301,723.
CNFN	GeneScaffold_967: at location 353190
XRCC1	GeneScaffold_968 at location 33,624-61,111.
APOE	GeneScaffold_1729 at location 58,701-60,486.
TOMM40	GeneScaffold_1729 at location 33,618-53,028.
**CEACAM homologs**	
ENSMICG00000004536	GeneScaffold_3524 at location 10,585-11,884.
ENSMICG00000015362	GeneScaffold_967 at location 469,227-484,984.
ENSMICG00000013460	Genescaffold_18018 at location 1,156-9,342.
ENSMICG00000003336	Genescaffold_24316 at location 584-8,193.
ENSMICG00000002461	Genescaffold_25774 at location 4,108-6,978.
ENSMICG00000002031	GeneScaffold_4201 at location 14,000-28,219.

Table S2. Inventory of CEACAM family genes in the opossum and the platypus.

Opossum chromosome 4			
**CEACAM homologs**	**Accession No.**	**Gene description**	**position (Kbp)**
Gnomon model: hmm52161			268816
MdoCEACAMI	XP_001373946	LOC100021942	368730
Gnomon model: hmm52469			369010
Gnomon model: hmm52931			369212
MdoCEACAMII	XP_001374110	LOC100022176	369223
Gnomon model: hmm53855			369311
Gnomon model: hmm54471			369417
MdoCEACAMIII	XP_001374175	LOC100022275	369293
MdoCEACAMIV	XP_001374551	LOC100022824	369727
MdoCEACAMV	XP_001374568	LOC100022853	369790
Gnomon model: hmm57551			369835
Gnomon model: hmm57859			369944
Gnomon model: hmm58013			370005
MdoCEACAMVI	XP_001374735	LOC100023084	370106
Gnomon model: hmm59091			370149
MdoCEACAMVII	XP_001374909	LOC100023335	370239
Gnomon model: hmm60323			370365
MdoCEACAMVIII	XP_001374992	LOC100023450	370398
MdoCEACAMIX	XP_001375006	LOC100023472	370543
Gnomon model: hmm60939			370558
MdoCEACAMX	XP_001375071	LOC100023563	370618
MdoCEACAMXI	XP_001375087	LOC100023589	370629
Opossum chromosome 2			
**CEACAM homologs**	**Accession No.**	**Gene description**	**position (Kbp)**
MdoCEACAMXII	XP_001362320	LOC100009812	102310-102287
MdoCEACAMXIII	XP_001363660	LOC100010509	102591-102599
MdoCEACAMXIV	XP_001363739	LOC100010542	102625-102707
MdoCEACAMXV	XP_001363998	LOC100010668	102783-102810
MdoCEACAMXVI	XP_001364388	LOC100010873	103071-103079
Platypus			
**CEACAM homologs**	**Accession No.**	**Gene description**	**position**
OanCEACAM16	XM_001518974.1	LOC100089680	Contig4712 at location 24,920-45,710.
OanCEACAM16LI	XM_001515791.1		Contig24216 at location 2,420-10,212.
OanCEACAM20LII	XM_001518997.1	LOC100089709	Contig2758 at location 24,075-32,457
OanCEACAM20LI	XM_001519009.1	LOC100089723	Contig2758 at location 35,563-61,899.

CEACAM family genes on unknown chromosome contigs of the opossum.

**CEACAM homologs**	**Gene description**
XP_001381920	LOC100033024
XP_001381900	LOC100032997
XP_001381842	LOC100032928
XP_001381705	LOC100032765
XP_001381428	LOC100032410

XP_001381421	LOC100032401
XP_001381408	LOC100032385
XP_001381402	LOC100032375
XP_001381400	LOC100032373
XP_001381170	LOC100032068
XP_001381138	LOC100032023
XP_001381135	LOC100032019
XP_001380988	LOC100031820
XP_001380977	LOC100031804
XP_001380959	LOC100031784
XP_001380833	LOC100031617
XP_001380831	LOC100031612
XP_001380820	LOC100031595
XP_001380816	LOC100031589
XP_001380797	LOC100031562
XP_001380790	LOC100031554
XP_001380772	LOC100031534
XP_001380753	LOC100031510
XP_001380745	LOC100031501
XP_001378114	LOC100027966
XP_001373697	LOC100021582
XP_001373624	LOC100021469
XP_001369443	LOC100015347

Table S3. Inventory of CEACAM family genes in nonmammalian vertebrates including, *X. tropicalis, D. rerio, G. aculeatus, T. nigroviridis,* and *T. rubripes* .

Clawed frog Xenopus tropicalis		
**CEACAM homolo**	**Gene description**	**Position**
XtrCEACAMI		scaffold_209:15,430-82,650
XtrCEACAMII	DN061666	scaffold_209:120,617-145,043
XtrCEACAMIII	EG655503	scaffold_209:458,121-742,786
XtrCEACAMIV	DT452251	scaffold_997:114,545-231,962
XtrCEACAMV		scaffold_998:63,633-100,454
XtrCEACAMVI	CF593042	scaffold_998:115,632-118,325
XtrCEACAMVII	CX796104	Scaffold_1867:9,152-14,026
XtrCEACAMVIII	AAI58512	scaffold_8873:3,751-13,999
Zebrafish Danio rerio		
**CEACAM homolo**	**Gene description**	**Position (Kbp)**
DreCEACAMI	LOC557409	chr16:12,319-12,360
DreCEACAMII	LOC798939	chr16:12,363-12,376
DreCEACAMIII	LOC557548	chr16:12,384-12,407
DreCEACAMIV	LOC799170	chr16:12,421-12,441
DreCEACAMV	LOC799237	chr16:12,444-12,471
DreCEACAMVI	LOC556295	chr16:12,484-12,520
DreCEACAMVII	LOC796823	chr16:40,178-40,479
DreCEACAMVIII	XP_001343594	chr16:59,500-61,500
DreCEACAMIX	LOC100004244	chr16:60,449-60,416
DreCEACAMX	LOC564690	chr19:5,753-5,782
DreCEACAMXI	BM534725	EST only
DreCEACAMXII	DN771854	EST only
Pufferfish Tetraodon nigroviridis		
**CEACAM homolo**	**Gene description**	**Position**
TniCEACAMIa	GSCT00014250001_prot	chr8:9,596,509-9600227
TniCEACAMII	CAF98056.1	chr8:875,570-883691
TniCEACAMIb	CAF93841.1	chrUn_random:10,186,988-10190617
Pufferfish Takufugu rubripes		
**CEACAM homolo**	**Gene description**	**Position**
TruCEACAMI	SINFRUP00000138632	scaffold_256 at location 89,696-98,074.
TruCEACAMII	SINFRUP00000144002	scaffold_256 at location 104,477-109,007.
TruCEACAMIII	SINFRUP00000144010	scaffold_256 at location 110,175-122,673.
TruCEACAMIV	SINFRUP00000155201	scaffold_35 at location 76,660-81,111.
TruCEACAMV	SINFRUP00000167512	scaffold_533 at location 35,056-38,511.

TruCEACAMVI	SINFRUP00000180360	scaffold_473 at location 7,856-9,519.
**Marker genes**	**Gene description**	**Position**
PAFAH1B3	SINFRUG00000130962	scaffold_256 at location 84,224-85,667.
XRCC1	SINFRUG00000138930	scaffold_35 at location 43,908-56,659.
Stickleback Gasteroteus aculeatus		
**CEACAM homolo**	**Gene description**	**Position**
GacCEACAMI	ENSGACP00000012415	chrXX:10,914,713-10,916,638
GacCEACAMII	ENSGACP00000012419	chrXX:10,916,140-10,918,317
GacCEACAMIII		chrXX:10,932,784-10,938,084
GacCEACAMIV		chrXX:10,940,522-10,945,509
GacCEACAMV		chrXX:10,954,154-10,956,659
GacCEACAMVI	ENSGACP00000025269	chrIV:21,600,952-21,603,332
GacCEACAMVII	ENSGACP00000025271	chrIV:21,603,623-21,605,250
GacCEACAMVIII		chrUn:46,108,025-46,113,427
GacCEACAMIX		chrUn:46,115,806-46,127,880
GacCEACAMX		chrUn:46,120,107-46,120,730
GacCEACAMXI		chrUn:46,125,575-46,125,640
**Marker genes**	**Gene description**	**Position**
TMEM145	ENSGACG00000003235	groupXX at location 629,982-640,598.
CNFN	ENSGACG00000009361	groupXX at location 10,890,611-10,891,256.
PAFAH1B3	ENSGACG00000009368	groupXX at location 10,901,665-10,911,782.
XRCC1	ENSGACG00000012505	groupXX at location 13,969,086-13,979,832.
ETHE1	ENSGACG00000012528	groupXX at location 14,018,982-14,023,948.

Table S4. PCR primers for the amplification of select CEACAM transcripts in tissues of the platypus, *T. nigroviridis* , and *D. rerio*.

Gene name	Primer nam	Sequences
**Platypus**		
OanCEACAM16	Forward	CAGGGCGCCTATACCTGCATCGCCAA
	Backward	CAGCGGCAACTGGCTGACTAGTCGAT
OanCEACAM16LI	Forward	CATCACACCAATCCATGATATTCAGA
	Backward	GTCCTGGAGCTCCACGGTGTTGGTGC
OanCEACAM20LI	Forward	CAACTTCATCATCAGTAATGGGAAAC
	Backward	CGGAGCCATTGTGAAACCAGTGATAC
OanCEACAM20LII	Forward	CATGTTACAGCACATGGCTGCCATTA
	Backward	GCTGTCTCTGCGCTGATCTGGACCTT
T. nigroviridis		
TniCEACAMIa	Forward	TGTCCATGCGCTGTCGGTAACAGAG
	Backward	TGGTATTGACGTCCTTCAGATCCTGA
TniCEACAMII	Forward	ATGTACATGACCTCGCCGTTTACCAT
	Backward	GGTTCTTCGCCACACAAGTATAGTTCC
TniCEACAMIb	Forward	TTCTCAAAGGTCAGGACTTCCTGCTGA
	Backward	AAAACTGTTTGAGGATAAAGTGACAG
D. rerio		
DreCEACAMI	Forward	AAGGATAACAGTCCTCTGTCTTCTAGC
	Backward	CCTCAATAGAAACACCATCTGGTCCAT
DreCEACAMVII	Forward	AACACCACAGTGACCTTCAGTTCATTG
	Backward	CAGAGACACTGCATTACATATCCAGAATG
DreCEACAMX	Forward	TTAACACTCACTTGCACCGCTGATTCG
	Backward	TGTGCATTGTAGGCTACACAGGTATAG

Table S5. PSG locus CNVs that were identified based on high-density probes (Conrad et al., 2010; Park et al., 2010).

CNVR	*start*	*end*	*length*	*type*	*CEU (%)*	*YRI (%)*	*Asian (%)*
CNVR7655.1	47464296	47465248	952	loss	5.0	0.0	
CNVR7656.1	47478751	47481362	2611	gain	5.0	0.0	
CNVR7657.1	47727125	47729754	2629	loss	0.0	15.0	
CNVR7658.1	48391645	48458439	66794	gain/loss	75.0	55.0	
CNVR7658.2	48000706	48238985	238279	loss	5.0	0.0	
CNVR7658.3	48157445	48183239	25794	gain	5.0	0.0	
CNVR7658.4	48197760	48327876	130116	loss	0.0	5.0	
CNVR7658.5	48239642	48492519	252877	gain	0.0	5.0	
CNVR7658.6	48308360	48340279	31919	gain	5.0	0.0	
CNVR7658.7	48319597	48329243	9646	gain	5.0	0.0	
CNVR7658.8	48351136	48392895	41759	loss	0.0	5.0	
CNVR_3824.1	47960658	48340565	379908				10.0
CNVR_3825.1	48381513	48461975	80463				83.0
CNVR_3825.2	48377689	48400480	22792				3.3

Table S6. A large proportion of CEACAM/PSG genes contain SNPs with high population differentiation.

Genes	*With gene region SNPs that have Fst at top 5% bracket*	*With nonsynonymous SNP that have Fst score at the top 15% bracket*	*Nonsynonymous SNP with Fst at the top 10% bracket*	*Frequency of the derived allele in (CEU, YRI, ASN)*
**PSG1**				
**PSG2**	●			
**PSG3**				
**PSG4**		●	**rs3859474**	**(0.63, 0.24, 0.06)**
**PSG5**				
**PSG6**				
**PSG7**				
**PSG8**				
**PSG9**				
**PSG10**				
**PSG11**	●	●	**rs10414166**	**(0.33, 0.00, 0.00)**
**CEACAM1**	●	●	**rs8110904, rs8111171, rs8111468**	**(0.01, 0.57, 1.00)(0.02, 0.44, --)***
**CEACAM3**				
**CEACAM4**	●			
**CEACAM5**				
**CEACAM6**				
**CEACAM7**				
**CEACAM8**	●	●	**rs8103051**	**(0.00, 0.33, 0.00)**
**CEACAM16**				
**CEACAM18**		●	**rs12610545**	**(0.03, 0.00, 0.38)**
**CEACAM19**	●			
**CEACAM20**	●			
**CEACAM21**	●	●	**rs714106**	**(0.23, 0.50, 0.88)**
**CEACAM22/FLJ41856**				
**CEACAM23/LOC114710**				
***, rs8111171 and rs8111468 have identical allele frequencies.**				
